# Testing theories of political persuasion using AI

**DOI:** 10.1073/pnas.2412815122

**Published:** 2025-05-02

**Authors:** Lisa P. Argyle, Ethan C. Busby, Joshua R. Gubler, Alex Lyman, Justin Olcott, Jackson Pond, David Wingate

**Affiliations:** ^a^Department of Political Science, Brigham Young University, Provo, UT 84602; ^b^Department of Computer Science, Brigham Young University, Provo, UT 84602

**Keywords:** persuasion, large language models, microtargeting, elaboration likelihood model, political communication

## Abstract

Despite its importance to society and many decades of research, key questions about the social and psychological processes of political persuasion remain unanswered, often due to data limitations. We propose that AI tools, specifically generative large language models (LLMs), can be used to address these limitations, offering important advantages in the study of political persuasion. In two preregistered online survey experiments, we demonstrate the potential of generative AI as a tool to study persuasion and provide important insights about the psychological and communicative processes that lead to increased persuasion. Specifically, we test the effects of four AI-generated counterattitudinal persuasive strategies, designed to test the effectiveness of messages that include customization (writing messages based on a receiver’s personal traits and beliefs), and elaboration (increased psychological engagement with the argument through interaction). We find that all four types of persuasive AI produce significant attitude change relative to the control and shift vote support for candidates espousing views consistent with the treatments. However, we do not find evidence that message customization via microtargeting or cognitive elaboration through interaction with the AI have much more persuasive effect than a single generic message. These findings have implications for different theories of persuasion, which we discuss. Finally, we find that although persuasive messages are able to moderate some people’s attitudes, they have inconsistent and weaker effects on the democratic reciprocity people grant to their political opponents. This suggests that attitude moderation (ideological depolarization) does not necessarily lead to increased democratic tolerance or decreased affective polarization.

## Significance

We leverage recent advances in AI in two original research studies regarding persuasion about controversial political issues in the United States. These studies make both methodological and substantive contributions to the study of persuasion. First, we demonstrate that large language models (LLMs) can be used to efficiently test theories of persuasion by overcoming many practical and logistical challenges faced by persuasion researchers. Second, we use LLMs to isolate and test the persuasive gains offered by two key hypothesized mechanisms of attitude change—message customization and elaboration. We show that LLMs are approximately equally persuasive across conditions and that LLM-generated messages with microtargeted customization and messages that promote elaboration through interaction are not clearly more persuasive than a generic message.

Persuasion is ubiquitous in social and political life. Significant efforts and resources of corporations, health professionals, advocacy groups, government organizations, political actors, religious leaders, and even coworkers, friends, and family members are devoted to persuading people to adopt different attitudes or behaviors, buy specific products, support particular political candidates or positions, conform with public health recommendations, comply with legal authorities, and more.

Persuasion is especially important in democratic political systems, where politicians, advocates, and policy outcomes rise and fall based on their ability to persuade potential supporters to their side. As some scholars have described it, “Democracy, in particular, is distinguished as a form of governance by the extent of persuasion relative to coercion” (1, p. 2). Although persuasion is essential for coalition-building and governance in democratic societies, it is simultaneously a source of significant concern: The possibility that demagogues might use inappropriate means of persuasion to manipulate mass opinion was a central concern in both Ancient Greece (2) and the American founding (3).

This long history of concern about and practice of political persuasion in democratic systems has gained renewed prominence in the public imagination in response to advances in AI, particularly generative Large Language Models (LLMs) such as ChatGPT, Gemini, Llama, and Claude. LLMs provide the possibility for mass production of persuasive political argumentation, commonly riddled with biased argumentation and misinformation (4). However, the magnitude of these concerns remains difficult to quantify, at least in part because rigorous scientific evaluation of the precise psychological processes that lead to effective persuasion (both human- and AI-produced) remains elusive, incomplete, and often contradictory (5). Research on persuasive messaging can struggle to observe persuasive interactions in natural environments, to fully isolate any single component of important social, communication, and psychological processes, and to separate theoretically meaningful variations from incidental features of a selected message or message sender.

We suggest LLMs can be applied at scale to produce real-time variations of arguments in a consistent tone, style, and format. This means that LLM tools can essentially operate like a very well-trained confederate in a lab or lab-in-the-field experiment, making them a useful, extremely cost-efficient tool for testing the effects of various different approaches to political persuasion. Using techniques refined in our previous work (6, 7), we prompt GPT-4 to use a range of different approaches to persuade study participants in the United States to adopt a different policy position on immigration (study 1) and K-12 education (study 2) than that with which they began. In our studies, GPT-4 interacts directly with human participants on the survey platform Qualtrics, where it pursues one of four different strategies for persuasion, or a control.

We demonstrate that LLMs can be a tool for evaluating persuasive strategies in interaction with human subjects at scale, empowering researchers to study persuasion more deeply and efficiently. Importantly, the integration of LLMs into a careful research design allows us to isolate features of the persuasive interaction—in this case, the amount of customization (8–15) and engagement with the message, also called “elaboration” (16–20)—while holding constant or generating random variance in other key aspects of the exchange.

We find evidence that all treatments result in significant attitude change averaging approximately 2.5 to 4 percentage points. Furthermore, we do not find evidence that either microtargeted message customization or interaction-induced elaboration leads to significantly more attitude change than a generically produced single message, and our design is well-powered to detect differences of 2 percentage points or more. Finally, we show that while the average respondent moderated their opinions on a political topic, bringing participants in closer issue proximity to their opponents, this did not typically lead to an increase in democratic reciprocity, by which we mean a willingness to see opposing views as reasonable and deserving of respect, while still disagreeing. This suggests that attitude change through political persuasion is likely to be an insufficient solution to the complex ills of affective political polarization that increasingly plague democratic societies today.

## Theory and Expectations

Drawing on existing research, we test two well-developed and widely deployed theories about what might make a message more persuasive: customization and elaboration.

One central theory of persuasion posits that a customized message—one that presents arguments adapted to the characteristics, values, or beliefs of the target—will be more persuasive than generic arguments (8, 9, 11). In a prominent textbook account of persuasion, Gass and Seiter claim that perhaps the most important lesson for successful persuasion is that “a persuader doesn’t move the receiver to the message, the persuader moves the message to the receiver” (21, p. 123). In the political realm, microtargeting is widespread (22, 23) and there is clear belief by campaign operatives and some research scholars that such efforts are effective (12, 13, 24). This has prompted some regulatory concern in both the United States (25) and Europe (26). However, other scholarly research on the effectiveness of microtargeting has produced more muted and conditional results (11, 14, 15, 27, 28).

Additionally, the Elaboration Likelihood Model (16, 17, 19, 20) suggests individuals are best persuaded via one of two routes which depend on an individual’s level of “elaboration,” or cognitive engagement with the persuasive message. In the central, or high-elaboration, route, individuals expend cognitive effort to evaluate the substance of arguments, and produce questions or counterarguments. Persuasion in this state is expected to be more substantial and durable than persuasion via the low-elaboration peripheral route, which often relies on passive attention and heuristic cues. Political communication research on elaboration suggests that inducing additional argument scrutiny or elaboration of political messages in experimental settings can foster increased persuasion in some cases but may also lead to increased resistance to the proposed argument (29, 30).

Practical research limitations have prevented previous research from fully isolating the psychological mechanisms of either customization or elaboration from other confounding social, psychological, or communicative aspects of a persuasive exchange. Specifically, in the predigital era, producing customization in a research environment required a human researcher to identify the relevant traits and produce or select an appropriate message for each respondent. For some scholarship, this meant customized messages were limited in scope and applied on the basis of predetermined decision rules, meaning that conclusions about the efficacy of customization were dependent on the quality of prewritten messages and matching processes. Alternatively, for human-produced customization, message construction added delay and introduced a human social element that made customized messages incomparable with generic messages for reasons other than just the customization of the content. New technological tools have helped overcome some of these limitations—in one application, Tappin et al. (11) combine message pretesting with machine learning to produce and select messages that are more persuasive to some demographic subsets of the population. Likewise, using an LLM to produce microtargeted messages, Hackenburg and Margetts (28) find little gain from customization, while other scholars (13, 31) find significant benefits from LLM-produced customization. We use an approach similar to this latter group of researchers, using an LLM to generate customized and uncustomized messages for respondents in different experimental conditions.

Scholarship on elaboration emphasizes that the probability of elaboration in any given case can be significantly affected by the motivation and ability of the message receiver, among other possible influences (17). Motivation and ability to elaborate are driven by both the individual’s own predispositions, including their personality and prior interest in the topic, and situational features, such as social norms, distractions, or expectation of response. Elaboration research in the political context thus often relies on heterogeneous effects among people who are expected to have varying motivation and ability to elaborate, such as political sophistication or need for evaluation (29, 32), rather than directly manipulating whether a given person engages in the psychological process of elaboration (but see ref. 30). Historically, experimentally manipulating respondents to engage in higher elaboration would require treatments that provide additional motivation or ability to respondents, and thus indirectly affect elaboration, or that require respondents to engage in a different set of tasks, interactions, or experimental contexts and, therefore, introduce other social or contextual changes. This means that the psychological process of elaboration itself has been remarkably difficult to isolate from other situational, source, or motivational factors that were incidentally comanipulated.

While existing research takes a variety of different approaches to increase elaboration, in our experiments, we test two different interaction-based persuasion strategies. In the first, we prompt the LLM to directly attempt to change the other person’s mind by providing reasons and argumentation. In the other, the LLM is prompted to engage in motivational interviewing. Theories of motivated reasoning (5, 33, 34) suggest that when presented with information that disconfirms one’s strongly held priors, individuals are motivated to counterargue in an attempt to retain those priors—an effect that can be amplified in high-elaboration persuasive contexts (17, 29). In some situations, this countermotivation can diminish, erase, or reverse the effects of the persuasive information. Motivational interviewing was developed in part as a way to mitigate this process, allowing individuals to identify things in their own experience and patterns of thought that allow for attitude change, rather than directly facing the discomfort of externally provided, challenging information. This method has proven quite effective in a variety of settings (35, 36), including as a companion to Cognitive Behavioral Therapy (37) in the treatment of mental illness.

One additional challenge for researchers of political persuasion is that, particularly in a polarized political environment, successful persuasion can mean several different things. Politically relevant attitudes and behaviors can include 1) attitudes about the content and justifications for support or opposition of particular policy positions or candidates, 2) attitudes about the groups of people who support or oppose (or who would be seen as the “winners” or “losers”) from the policy or election—such as political parties or racial groups, or 3) the actual behaviors taken to advocate for the policies or candidates—such as voting. Prior research finds that these different attitudinal and behavioral changes are not always in sync. For example, political microtargeting research by Dobber and coauthors (14, 15) finds different outcomes for dependent variables of candidate support, party support, vote intention, and actual votes.

In the United States, polarization research clearly demonstrates a rise in affective polarization (or polarization based on feelings about partisan groups) (38, 39), but is more conflicted about whether these divides are spurred by significant policy disagreement (40, 41) or whether attitudes about the actual policies have themselves grown more polarized (42, 43). This ongoing debate signals that it is not a given that shifting any one component of an individual’s politically relevant conclusions in this framework (attitudes about policy, attitudes about groups, or political behavior), will necessarily impact either of the other attitudes/behaviors. Indeed, in our prior research (7), we demonstrate that LLM-assisted political conversations can increase democratic reciprocity (one measure of outgroup respect and tolerance), even if no issue-based persuasion occurs and conversation participants do not close any ideological distance.

Prejudice reduction, or changing negative attitudes and stereotypes about groups, is the focus of a vast interdisciplinary literature that generally finds mixed results; it is remarkably difficult to impact attitudes toward groups (44, 45). By contrast, research focused on issue-based attitudinal change by Coppock (46) and others (47–50) suggests that compelling political information generally persuades its targets, but only in the absence of strong group cues regarding the identity of the sender. They find the effects of such persuasive information are often small but are consistent and durable. Building on this work, we design a study in which the LLM messages do not provide explicit group cues, and our primary focus is changing policy-relevant political issue attitudes. As secondary outcomes, we also measure self-reported voting intention (an imperfect proxy for behavior), and democratic reciprocity toward one’s political opponents (as a measure of group-level prejudices).

Based on this past work, we propose the following two hypotheses:

**H1**: All LLM-generated persuasive treatments will significantly move policy attitudes in the direction of their argument in comparison to the control.

However, the research reviewed here suggests that the magnitude of these effects, relative to our control conditions, should vary depending on the characteristics of the appeal. Participants in our study are randomly assigned to one of four different types of treatment messages: 1) a one shot message that presents strong, but generic arguments, 2) a one shot microtargeted message that draws on information gathered at the start of the survey to make the strongest argument for a given participant, 3) an interactive, 6-turn conversation during which the LLM directly presents strong arguments to persuade, or 4) an interactive 6-turn “motivational interview” with participants to encourage them to change their position. Our second hypothesis has to do with the ordering of the magnitude of these treatment effects:

**H2**: Relative to the control conditions, we expect the effect sizes of the four strategies to be the following, from smallest to largest: one shot generic message, one shot microtargeted message, interactive direct persuasion, and interactive motivational interviewing.

As previously noted, in addition to persuasive effects on people’s relevant attitudes, we consider respondents’ political support for a candidate and democratic reciprocity—their respect and tolerance for people who hold the opposite view. Given the mixed results on the connection between attitudes and group perceptions discussed earlier, we were uncertain about the direction of treatment effects for these outcomes.

Additional details about the preregistration of hypotheses and design for both studies can be found in *SI Appendix*, section 1.3.

## Design

Past research has demonstrated that LLMs are highly capable of producing messages that are comparably persuasive to those produced by human authors (31, 51–54). Building on this work, we leverage the near-instantaneous generative and persuasive capacity of LLMs in a Qualtrics-based survey experiment. Respondents were randomly assigned to one of six conditions (two controls and four treatments; see *Materials and Methods*). All the message content was presented to respondents in the context of the survey experience, which creates a comparable experimental environment, social context, and motivational state for all respondents across treatment and control groups. Additionally, the use of the LLM allows each of the respondents to receive a slightly different one-shot message, or set of messages, such that the basic content, objective, and tone remain constant but the specific wording is not identical between subjects. Among communications and persuasion scholars, this approach is a best practice to test different types of approaches to persuasion, as the results are then not driven by the incidental particulars of any one researcher-selected message (18, 55).

To evaluate our hypotheses, we designed, preregistered, and fielded two experiments in May of 2024 (Study 1 n = 1,862, Study 2 n = 1,819). The studies focused on two divisive and salient political topics in the United States: immigration in study 1 and K-12 education in study 2. We chose immigration due to its consistent importance in American (and global) politics (56, 57), sustained interest in this topic among social scientists (58–63), and clear divisions between political groups in the United States (56, 64, 65). In study 2, we focused on the role of teachers’ personal social and political views in the K-12 classroom for similar reasons: It is highly divisive in the United States (66) and the topic of many recent political debates at the national and local level (67–69). Additionally, we expected partisan attitudes on this issue to be the inverse of those for immigration: Immigration attitudes are associated with higher levels of moral conviction and less flexibility for liberals (70), whereas conservatives are more vocally supportive of government action and restriction in the space of K-12 education curriculum (71).

## Results

We begin by first showing that the LLM-produced persuasive messages across conditions functioned as expected, such that the messages in each type of treatment 1) were differentiated in presentation from other treatments so the treatments are distinct, but 2) were similar in the content of the arguments they made, such that we do not have significant differences in argument quality across treatments. To explore this, we use machine learning techniques to generate visualizations of the AI-generated texts in two ways. First, we transformed the complete text the LLM wrote for each survey respondent into a 1,024-dimensional vector of semantic features through a technique known as embedding; these vectors were then projected down to two dimensions and plotted. Importantly for the purposes of the visualization, no information about the subject’s assigned condition was included in the embedding and projection process; the colors and glyphs were added after analysis to distinguish the ideological direction and treatment condition for interpretation.

**Fig. 1.**
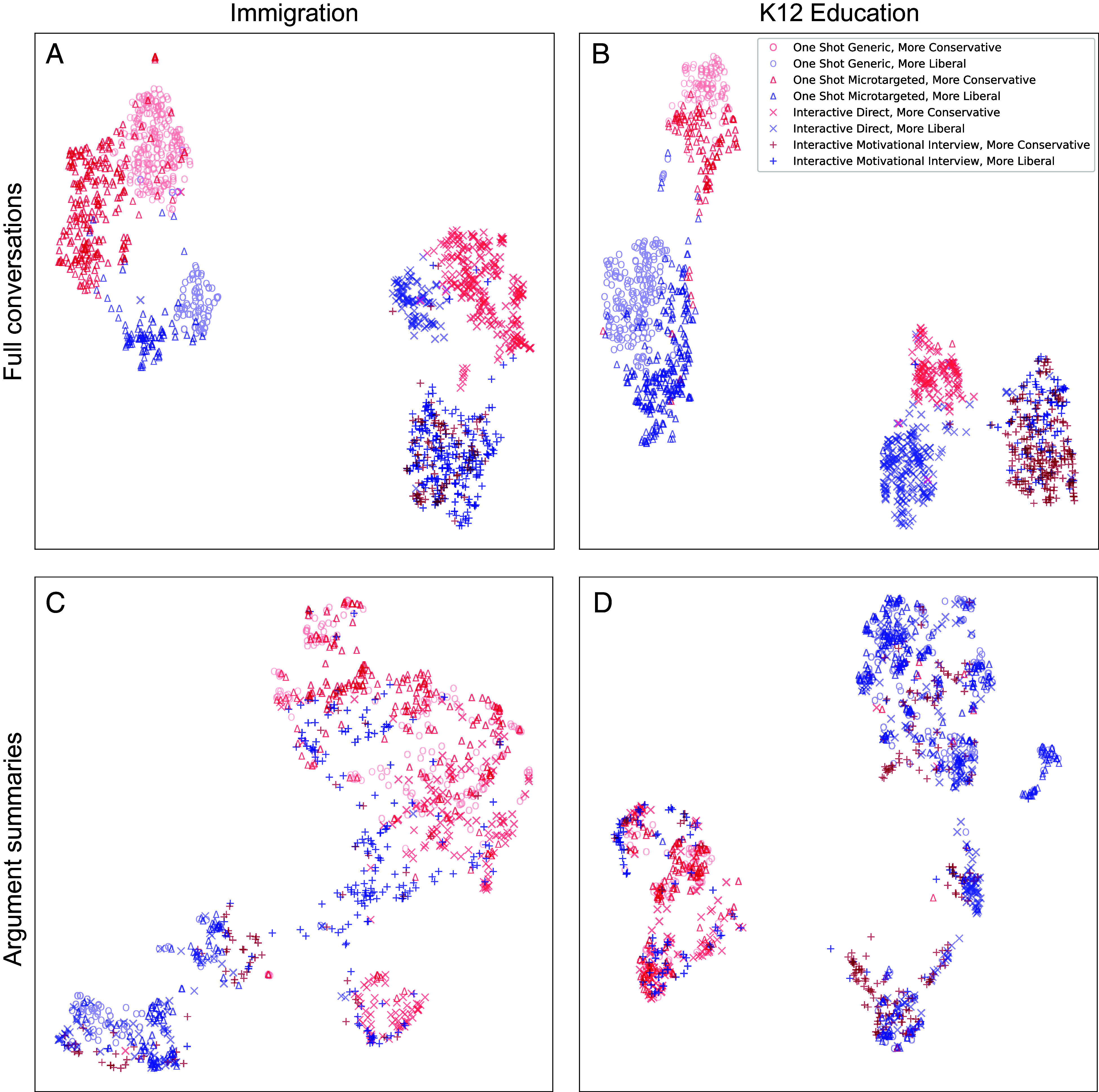
Distribution of embeddings of text written by the AI in the different experimental conditions. Axes are not interpretable. Each point represents an embedding of the text written for a single subject. The *Top* row shows the embeddings of the full transcript of text generated by the LLM for the respondent, with the Immigration study in panel *A* and the K12 Education study in panel *B*. These panels show evidence that the persuasive approach meaningfully differed across treatment conditions. The *Bottom* row shows the embedding of a one-sentence GPT-4o generated summary of the main argument made by the LLM for each respondent, with the Immigration study in panel *C* and the K12 Education study in panel *D*. These panels show evidence that the core content of the position and arguments were not notably different across treatment conditions. See *SI Appendix* for additional analysis details.

The two panels in the *Top* row of Fig. 1 (panels *A* and *B*) present the results. While the two dimensions that form the axes of the panel are not directly interpretable and may not be directly comparable across the two studies, these panels provide evidence that the text generated for each treatment condition appears to be more similar within treatment than across treatments. The *Left* of each panel clusters the one-shot messages; text generated for one-shot messages in generic (circle) and microtargeted (triangle) conditions occupy a similar semantic space but in distinct clusters from each other. To the *Right* of each panel, the interactive messages cluster together, indicating they were more similar to each other than to the one-shot messages, but are quite distinct from one another. Moreover, arguments in a more conservative direction (red) are more similar to one another than arguments in a liberal direction (blue). The exception to this is the motivational interviewing condition, in which there is very little differentiation between liberal and conservative positions; this is not unexpected, as the LLM in the motivational interviewing condition did not provide any new information or argumentation, and instead solicited and responded to whatever respondents themselves brought to the issue.

Panels *C* and *D* of Fig. 1 provide evidence that the LLM-produced persuasive approach does not incidentally covary with argument content. These panels are based on a summary of the core argument, rather than the full text of the treatment messages. Specifically, we prompted GPT-4o to generate a one-sentence summary of the core argument being made by the LLM in the generated text for each respondent. This approach allows us to separate content from style. These summaries of content were then embedded and visualized in the same way as the full text of the arguments. Here, we see that, aside from expected distinctions between more liberal and more conservative arguments, the content of all of the conditions overlaps strongly. Thus, Panels (*A* and *B*) show that the presentation of the arguments differed across conditions and direction, while Panels (*C* and *D*) show that the content of the arguments was largely the same across conditions, but different across direction, as we would hope. These findings suggest high treatment internal validity. Additional analysis of the content of the conversations is available in *SI Appendix*.

**Fig. 2.**
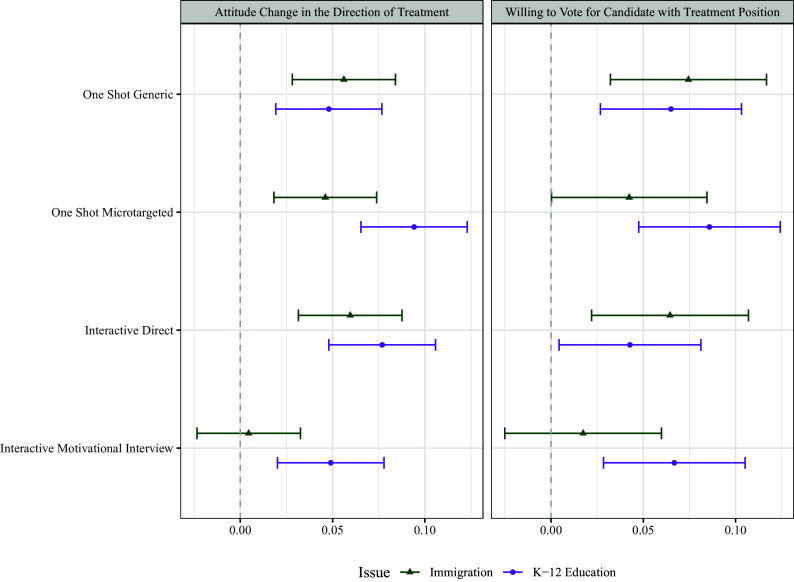
Experimental Results Coefficient Plot. Point estimates are coefficients from an OLS model, with no additional control variables. Errors are 95% CIs. Baseline is composed of the combined static and interactive control conditions. The full potential scale of the dependent variable of attitude change ranges from −1.33 to 2; the effects shown here represent approximately 0.05 to 0.08 unit, or 2.5 to 4 percentage point, changes. With the exception of motivational interviewing on the immigration topic, all four types of persuasive AI produce significant attitude change relative to the control (*Left* panel, pre–post difference) and shift vote support for candidates espousing views consistent with the treatments (*Right* panel; post-only). However, we do not find evidence that message customization via microtargeting nor cognitive elaboration through interaction with the AI have demonstrably more persuasive effect than a single generic message.

We next evaluate the extent of attitude and intended behavioral change as a result of the treatment interventions in two ways. First, respondents were asked three questions about their attitude on the policy issue (immigration or K-12 education) both before and after receiving the treatment intervention (see *Materials and Methods* for more detail). The *Left* panel of Fig. 2 depicts the pre/post change in policy attitudes for respondents by treatment condition as a marginal effect (estimated using OLS, with no covariates beyond the treatment indicators) relative to the pooled control conditions. These effects represent pre/post-treatment change in attitudes in the direction of the treatment, meaning that positive values indicate movement in the direction of the treatment text and away from the respondent’s pretreatment attitude. The plots show results for both the immigration study and the K-12 education study. The *Right* panel of Fig. 2 shows similar marginal effects for a posttreatment-only variable asking respondents their willingness to vote for a political candidate who took a position consistent with the direction of the treatment they received.

In support of our first hypothesis, Fig. 2 shows consistent persuasive effects (change in attitudes in the direction of the treatments and away from the respondents’ own pretreatment positions) in both studies, for both the attitudinal and behavioral intent outcome measures. The exception to this is the interactive motivational interviewing condition in the immigration experiment. In general, however, the treatments generated the kinds of persuasive effects other work would predict (46) in a context of reasoned argumentation without explicit group cues.

As expected, these treatment effects are relatively small in magnitude. The attitude change metric is coded such that positive values indicate movement in the direction of the treatment position and negative values indicate movement away from the direction of treatment [due to the documented potential for backlash effects (72)]. The total possible change for any given respondent is 2 points, but because of their different pretreatment starting points, the full scale ranges in theory from −1.33 to 2. In practice, we observe a range from −1 to 1.33 in our data. As such, the marginal effects represent approximately 0.05 to 0.08 unit changes, which correspond to 2.5 to 4 percentage point changes.

The vote choice metric is coded from −1 to 1 and shows between-subjects effects of similar magnitude to the average attitude change. These effect sizes are consistent in magnitude to those reported in human-based persuasion studies taking a similar approach (46); like these other studies, it is possible the magnitude of these effects might shrink in a real-world context, where individuals encounter this type of messaging surrounded by more distraction (73). It is also unclear what effect sizes might look like with repeated and longer exposure; participants in our studies had just one interaction with treatments that lasted anywhere from seconds to a few minutes at most. These considerations merit further study.

Interestingly, we find no support for our second hypothesis regarding the ordering of the magnitude of treatment effects. Generally speaking, the one shot generic message, one shot microtargeted message, and interactive direct persuasion conditions all generate persuasive effects of similar magnitude. Motivational interviewing, in contrast to our expectations, often has the weakest and least consistent persuasive effects on both attitude change and vote support. We designed our studies to ensure sufficient power to detect small differences across treatments, and our post hoc power analysis suggests sufficient power to detect relatively small differences between treatment conditions (effects larger than about 1.5 percentage points; see *SI Appendix*, section 7.4). While that analysis shows that we may not be able to reliably detect very small differences (e.g., 1 to 1.5 percentage points), the design we use here has sufficient power to detect persuasive effects of the magnitude usually hypothesized by theories of customization and elaboration. In other words, while there may be very small differences between these treatments that we cannot reliably identify, for these differences to matter, they would have to be much smaller than generally expected, in the same direction, and very similar to one another.

We think it substantively important that both the generic and microtargeted one-shot messages, on average, produce attitude change of roughly the same magnitude as each other, and as the dynamic treatment. This adds to a growing body of evidence showing that, despite many reasonable concerns, microtargeting by LLMs does not appear to produce significantly more effective persuasive messages than other information-generating processes (11, 28, 74) c.f. refs. 31 and 12). This does not suggest that AI customized messages are not persuasive, just that the customization itself does not seem to provide strong persuasive advantages. Moreover, that the one-shot messages seem to be as persuasive (or more, in some cases) than the approaches leveraging interaction to promote elaboration suggests that those interested in pursuing persuasion have a much simpler path than engaging participants in interactive conversations. Again, this is not to say that conversational approaches do not have a significant effect in the right circumstances (75), but rather that the value of these approaches might come from other social, motivational, or source cues rather than directly from the psychological process of elaboration. We purposefully focused on short conversations in our interactive conditions as these are most common in the mode of our experimental delivery; results might vary with a different approach that emphasizes longer conversations in different contexts.

While all subjects in the study received counterattitudinal messages, we also consider the effects of these persuasive treatments on subjects with different initial opinions on the topic of the study (that is, those with initially more conservative views on immigration, more liberal views on K-12 education, etc.). The full set of results that explore this potential variation and their corresponding sample sizes can be found in *Materials and Methods* of this paper (see Fig. 4 and Table 1). These analyses reestimate the results in Fig. 2 broken out by initial views on the topic in the messages/discussions; in these comparisons, we find striking homogeneity across all arms in these different subgroups. By this we mean that although there are sometimes subgroup differences in the level of persuasiveness of all treatments, the difference between treatments for a particular subgroup is generally not significant.

**Table 1.** Number of participants, grouped by pretreatment attitude and condition

	Immigration			K-12 education		
	Conservative	Liberal	Neutral	Conservative	Liberal	Neutral
Interactive control	143	85	83	148	62	98
Interactive direct	147	88	82	158	68	84
Interactive motivational interview	148	84	86	159	51	100
One shot control	136	85	86	157	68	83
One shot generic	144	80	93	163	49	95
One shot microtargeted	153	86	79	166	63	83

**Fig. 3.**
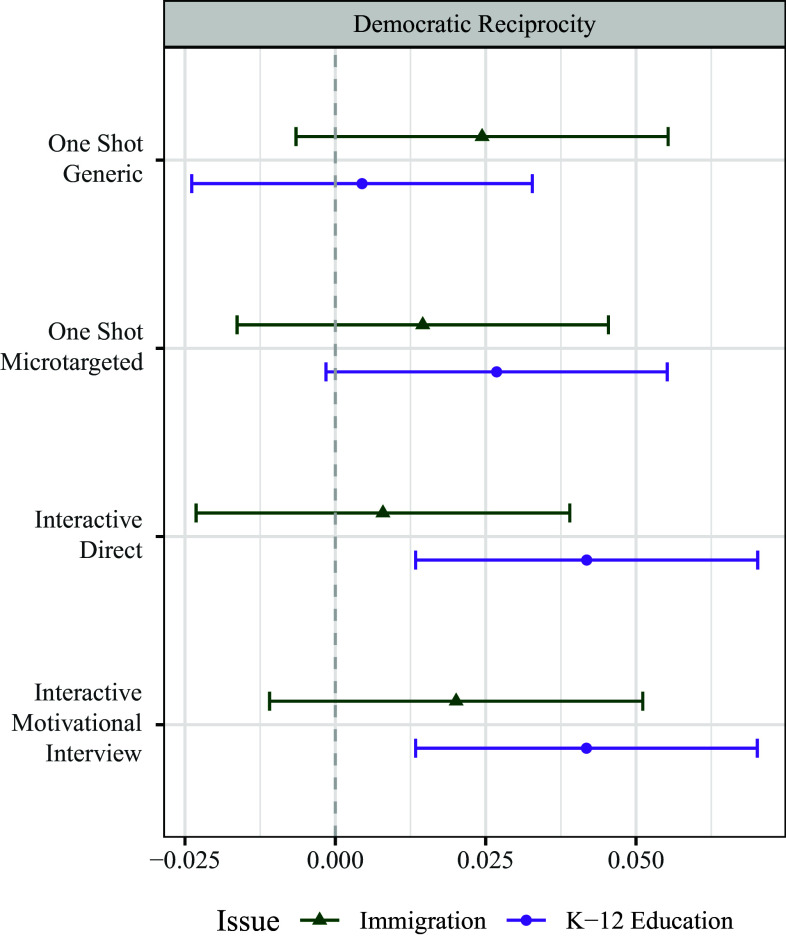
Democratic Reciprocity Results. Point estimates are coefficients from an OLS model, with no additional control variables. Errors are 95% CIs. Baseline is composed of the combined static and interactive control conditions. We find that persuasive messages have inconsistent and weaker effects on the democratic reciprocity people grant to their political opponents. This suggests that attitude moderation (ideological depolarization) does not necessarily lead to increased democratic tolerance or decreased affective polarization.

Finally, we turn to a discussion of results related to our last dependent variable: democratic reciprocity. To generate this outcome variable, respondents were asked about their willingness to see people who disagree with them on the target issue as reasonable, as well as a measure of respect for such individuals. As illustrated in Fig. 3, we see smaller and more contingent effects of the persuasive treatments on this outcome measure. Particularly in the One Shot conditions and on the issue of immigration, in spite of significant ideological attitude moderation, respondents were not more willing to change their perceptions of their opponents. In other words, although the ideological distance between respondents and their opponents decreased, they did not find the people on the other side more reasonable or worthy of respect than previously.

The only conditions in which there is a significant increase in democratic reciprocity are the interactive chat conditions on the issue of K-12 curriculum. We suggest one possible cause of the increased democratic reciprocity in the interactive conditions of the K-12 curriculum study is that tolerance of diversity is an important argument made by the LLM in many of these interactions (*SI Appendix*, section 8); therefore, tolerance in the K-12 education study is not only an ancillary effect of persuasion but also a core substantive element of the persuasive messaging. Regardless, whatever the underlying process, we find that issue-based attitude change is only contingently and intermittently connected to reducing group-based prejudices and increasing tolerance for disagreement.

## Discussion and Conclusion

In prior research, our team demonstrated that LLMs can be used to improve the tone of politically fraught conversation, leading to improvement in participants’ democratic reciprocity that is not dependent on a change in their policy attitudes (7). Here, we demonstrate the inverse: that LLMs can generate persuasive messages that move individual issue positions in more moderate political directions, but that this may not move fundamental democratic attitudes about those with whom they disagree. Thus, even though people may come to find more common ground on the core content of the policy issues, that does not guarantee that they will be willing to engage in deliberative exchange or extend respect to those who hold differing views. This suggests that resolving important polarizing divides in our political landscape may require something beyond each side delivering better arguments to narrow the ideological divide between opposing camps.

We reach this conclusion through an examination of the effects of four different persuasive tactics, varying in their customization and induced level of elaboration, on attitude change, vote support, and democratic reciprocity. Our results suggest LLM-generated persuasive texts can indeed move people’s policy positions but that customization and elaboration as features of these messages may not provide much added persuasive impact over a one-shot generic message generated by the LLM. It may be that there are some small gains to be had from these methods, but they would need to be very small (and smaller than expected by researchers of persuasion) to be consistent with our results.

What do these findings suggest about these prominent theories of persuasion? At the very least, they support dissenting voices on the persuasive impact of either customization or elaboration (11, 28, 74, 76, 77). They raise the possibility that these established and influential theories may be in need of replacement or at least revision. We fail to find the evidence that these theories would have predicted, as the persuasive effects of more elaborative discussions and customized messages appear indistinguishable from a more generic persuasive appeal. Indeed, our results mirror those from Coppock (46) and others whose work suggests that the content quality of persuasive arguments is more important than either elaboration or customization.

However, we urge caution in overinterpreting these results. In these studies, we focus on specific parts of these theories—(1) message tailoring based on a specific set of demographic and political characteristics and (2) chat-based conversation as a route to elaboration. This choice allows us to leverage the strengths of LLMs as research tools and to confidently reach conclusions about these elements while holding constant or providing stochastic variation in other elements of the persuasive interaction. However, there are other kinds of customization and other routes to elaboration that we do not explore. These results cast doubt on these specific approaches to elaboration and customization while leaving room for other approaches to be more important in persuasion.

For example, it may be that there is some piece of difficult-to-obtain background information that would promote more attitude change over the more generic approach we have taken here. Or, it is possible that background information is only impactful when used in interpersonal human interaction—not because of the change to the information but because of the interpersonal bond it creates between persuader and target. Additionally, a face-to-face experience with another person might engage additional social pressures and interpersonal dynamics related to elaboration and persuasion, beyond just the cognitive investment in the arguments. Elaboration effects might also depend on greater internal motivation than might be found in a randomized experiment like ours. Repeated, overtime engagement with a persuasive tactic may also promote more attitude change and fall more in line with the ideas of the Elaboration Likelihood Model.

Regardless, our results suggest some need for additional theorizing, caveats, or bounds, on the effects of customization and elaboration, and suggest LLMs as a viable tool to systematically explore many of these bounds. Given our results, if future work shows evidence for the effectiveness of other approaches, it should also provide an explanation for why those approaches, and not the more common ones we consider here, operate differently, especially given that theories of elaboration and customization as currently articulated do not anticipate such differences.

Beyond the immediate benefits of these results to researchers and political practitioners, we suggest the project itself usefully illustrates advantages of generative AI for social science research, and specifically for the study of persuasion and communication. In these studies, an LLM allowed us to randomize thousands of people to experimental conditions testing carefully controlled and varied approaches to political persuasion using custom messages produced in real time. Absent an LLM, such a task would require significant additional logistical and human costs, and would introduce other potential confounds into the experimental design. Our conclusion here is not that LLMs provide a replacement for every conceivable persuasive tactic—for example, discussions among close personal connections—but that they can significantly advance the study of critical theories of political persuasion when implemented thoughtfully.

## Materials and Methods

We fielded two survey-based experiments through CloudResearch, constructing samples that roughly matched the US census on gender, age, and race/ethnicity. We also ensured that the sample contained an even balance of Republicans and Democrats (as well as a smaller amount of political Independents). We chose this particular vendor (CloudResearch) on the basis Stagnaro et al. (78), who systematically compared competing online survey research platforms. We wanted to maximize survey representativeness and respondent attentiveness. *SI Appendix*, Table S1 describes the sample from this study. Respondents provided informed consent and were paid $3 for their time. This study was approved by the Office of Human Research Protections at Brigham Young University, under protocol IRB2023-420.

The entire experiment for both studies occurred within Qualtrics. Respondents first consented to participate and completed a reCAPTCHA task. They then indicated how important the topic of the treatments (immigration or K-12 education) was to them and reported their general level of political interest. We next measured respondents’ pretreatment attitudes on the issue with a set of three questions, drawn from or adapted from the ANES, Pew Research, and other sources.

For immigration, we asked whether respondents favored “increasing or decreasing government spending to secure the border” (on a seven-point scale from “increase a great deal” to “decrease a great deal,” reverse coded), whether they thought “the number of immigrants from foreign countries who are permitted to come to the United States to live should be increased or decreased” (on a seven-point scale from “increase a great deal” to “decrease a great deal”), and whether they favored or opposed “changing government regulation on businesses to make it easier to sponsor immigrant visas” (on a seven-point scale from “favor a great deal” to “oppose a great deal”). Factor analysis supported aggregating these items into a single average. The factor loadings were, respectively, 0.5, 0.91, and 0.75.

For K-12 education, we asked whether respondents favored “giving parents more control in deciding which controversial social and political topics are taught in public school?” (on a seven-point scale from “increase a great deal” to “decrease a great deal,” reverse coded), whether they thought “the government should do more or less to prevent teachers from bringing their social and political views into the classroom” (on a seven-point scale from “much more” to “much less”), and whether they thought public schools should “increase or decrease the amount of time spent teaching current social and political topics that might be controversial” (on a seven-point scale from “increase a great deal” to “decrease a great deal”). Factor analysis again supported aggregating these items into a single average. The loadings were, respectively, 0.77, 0.61, and 0.67.

In both studies, respondents then completed a set of demographic questions on their age, gender, race/ethnicity, religion, marital status, income, occupation, education, city and state, partisanship, and political ideology. They were then presented with a question intended to measure their attentiveness (79, 80).

At this point, respondents were randomly assigned to one of six conditions. Each involved an experience with an LLM-generated message in some form, although in different ways and to different degrees, as explained below. While the control conditions were meant to provide an experience orthogonal in its impact on our outcome variables, every treatment attempted to persuade respondents away from their pretreatment attitudes on immigration/K-12 education. Due to a programming error, in study 1, all respondents with a neutral pretreatment position received a message supporting increased immigration. In study 2, this error was corrected and those with neutral pretreatment policy views were randomly assigned to either pro- or anti-school regulation treatments.

As noted in the previous section, we consider persuasive tactics that involve different levels of customization and depth of processing. Our four treatments are as follows; the specific prompts provided to the LLM and a description of our prompt engineering process can be found in *SI Appendix*, section 2.

- One shot generic message: We prompted GPT-4 to generate what it considered the most persuasive message on the topic at hand. This treatment uses an LLM to generate a single message, uncustomized to the respondent, designed to persuade the respondent to change their views on immigration/K-12 education. The message is generated for each respondent but does not incorporate any respondent characteristics in the generation process. In the prompts for this treatment, the LLM was instructed to take on the role of an expert in crafting persuasive messages for the public and given a set of steps to work through in creating the message with the goal of persuading the respondent.
- One shot microtargeted message: This treatment uses an LLM to generate a single message, customized to the respondent, designed to persuade the respondent to change their views on immigration/K-12 education. The message is generated for each respondent and is customized to the respondent based on their background demographics and views. This is done by giving the LLM the respondents’ answers to the pretreatment demographic questions in the prompting window. In the prompts for this treatment, the LLM was instructed to take on the role of an expert in crafting persuasive messages for specific people and told to tailor the message to the specific individual. The LLM was given the background of the respondent but instructed not to explicitly bring up these characteristics. As in the uncustomized treatment, the LLM was given a set of steps to work through in creating the message with the goal of persuading the respondent.
- Interactive direct persuasion: This treatment uses the LLM as a dynamic interviewer applying direct persuasion tactics to encourage attitude change by providing arguments supporting the position on immigration/K-12 education that goes against respondents’ preexisting views. Respondents in this condition have a 6-turn conversation with the LLM, which dynamically responds and asks follow up questions. Respondents were required to engage in the conversation for six turns before proceeding and were not allowed to continue the conversation after these turns. The LLM was prompted to use various tactics of successful persuasion drawn from existing research (81, 82) and to mimic citizens’ ordinary attempts at persuasion. The conversational interaction is intended to increase the depth of processing (elaboration) that respondents experience in this condition. The prompts for this condition gave the LLM the role of an expert in psychology and persuasion with the goals of presenting the strongest argument as possible on the assigned stance to persuade voters. The LLM was given the background of the respondent, instructed not to explicitly bring up these characteristics, and presented with a series of tactics or approaches to take in the conversation. The LLM received the full conversation transcript in each API call for the next response.
- Interactive motivational interview: This treatment uses the LLM as a dynamic interviewer, applying the tactics of motivational interviewing to encourage reflection and attitude change by bringing to light the respondents’ existing competing beliefs and lived experiences (35, 36). Respondents in this condition have a 6-turn conversation with the LLM, which dynamically responds and asks follow up questions. Respondents were required to engage in the conversation for six turns before proceeding and were not allowed to continue the conversation after these turns. The prompts for this condition gave the LLM the role of an expert in motivational interviewing with the goals of persuading voters on the topic of immigration/K-12 education by helping respondents to convince themselves of the assigned stance. The LLM was given the background of the respondent, instructed not to explicitly bring up these characteristics, told not to introduce new arguments, and presented with a suggested motivational interviewing sequence of questions and reflections to work through in its responses. The LLM received the full conversation transcript in each API call for the next response.

Participants were also randomly assigned with equal probability to one of two control conditions. Following our preregistration plan, we considered if these conditions were statistically different from each other on our key dependent variables; they were not (*SI Appendix*, section 6.1). As such, in our analyses, we pool respondents from these two control groups together to form the baseline condition for all subsequent analyses:

- Interactive control: This treatment is a dynamic 6-turn conversation with the LLM on the topic of board games, not intended to persuade or raise the topic of immigration or K-12 education. Respondents were required to engage in the conversation for six turns before proceeding and were not allowed to continue the conversation after these turns. The prompts for this condition told the LLM to take on the persona of a board game expert and to ask open ended questions about the respondent’s feelings about board games. No demographic information about the respondent was included in this prompting.
- One shot message control: This treatment is a static single, uncustomized message generated by the LLM on the topic of board games, not intended to persuade or raise the topic of immigration. The message is the same for all respondents and does not incorporate any respondent characteristics in the generation process. *SI Appendix*, section 2 contains this message.

In *SI Appendix*, section 7.4.2, we report the average length of these conversations. We observed similar conversation lengths across the two studies. After these treatments, respondents were again asked the same three policy questions they were asked pretreatment. This within-subjects measurement strategy follows recent methodological conclusions in political science about asking respondents the same questions before and after experimental treatments (83). Participants then answered questions gauging their confidence in their views on immigration/K-12 education (on a five point scale from “not at all confident” to “very confident”), how likely they would be to vote for a candidate espousing the view they received in their treatments (on a five-point scale from “not at all likely” to “very likely”), how persuasive they felt the treatment they received was (on a five-point scale from “not at all persuasive” to “very persuasive”), and then four questions to measure democratic reciprocity—a willingness to listen to and engage with political opponents—drawn from our prior work (7). Finally, we asked respondents who they thought created the treatment they received (with answer choices including a variety of different people and also a chatbot) and the degree to which they were concerned or excited by AI [drawn from research by the Pew Research Foundation (84)]. At this point, participants were debriefed and the study concluded. Throughout this study, we observed very low levels of drop-out or attrition. This suggests to us that issues of differential attrition by treatment conditions are not influencing the results presented here. See *SI Appendix*, section 9 for additional details on attrition at various points in the design.

*SI Appendix*, Fig. S1 graphically illustrates the design of these studies. Further details on all points can be found in *SI Appendix*.

**Fig. 4.**
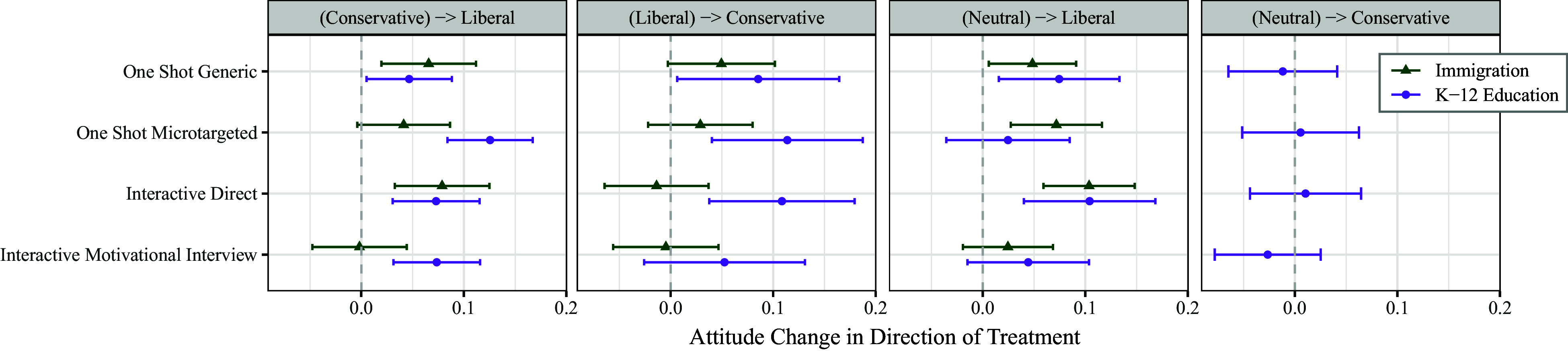
Treatment Effects by the pretreatment attitude (in parentheses) and the direction of the treatment condition.

All respondents received a treatment in the opposite direction as their initial attitudes. Respondents with an initially neutral attitude were randomly assigned to treatments in either direction. However, due to an error in programming, all neutral respondents in the first (immigration) study received a treatment in the liberal direction. As Fig. 4 indicates, this did not affect our results. Table 1 shows the number of respondents with conservative, liberal, and neutral pretreatment policy positions assigned to each treatment in both studies.

Fig. 2 in the main text presents the main treatment effects combining across these pretreatment attitudes (which we preregistered as our primary and first comparisons). Fig. 4 breaks this out further to show the treatment effects by groupings of the respondent’s pretreatment attitudes.

These results show no significant differences between the treatment conditions within any subgroup of the study, meaning that the different persuasive approaches do not appear to interact with the topic to produce more persuasion in some cases than others.

However, there are some intercept-shift differences for some of the subgroups or on some of the topics. This is most apparent in the second panel, where the LLM was more persuasive in persuading those with liberal policy positions to a conservative position on K-12 education than on immigration. There are multiple potential mechanisms for these kinds of differences: The LLM might produce messages that are more persuasive for some positions on some topics, individuals who hold some positions might be more set in their views and resistant to persuasion, etc. As this was not the focus of these studies, the design and data generated in these studies leave us unable to distinguish between competing explanations. As such, we report results on aggregate effects.

Replication data and code files can be found at https://osf.io/kgqrn/ (71).

## Supplementary Material

Appendix 01 (PDF)

## Data Availability

Anonymized survey data have been deposited in OSF (https://osf.io/kgqrn/) (71).
