# Supplementary material for: Testing theories of political persuasion using AI
Source: Proc Natl Acad Sci U S A. 2025 May 2;122(18):e2412815122. doi: 10.1073/pnas.2412815122 (PMC12067286; doi:10.1073/pnas.2412815122)
Supplement: Supplementary file 1 — Appendix 01 (PDF) [file pnas.2412815122.sapp.pdf]

Testing theories of political persuasion using AI

Argyle, Busby, Gubler, Lyman, Olcott, Pond, Wingate

April 16, 2025

Contents

1 Survey Data Collection **2**

1.1 Sample Demographics . . . 2
1.2 Visualization of Research Design . . . 2
1.3 Pre-Registration . . . 3
1.4 Immigration Survey Instrument . . . 3
1.5 K-12 Education Survey Instrument . . . 7

2 LLM Prompts to Generate Treatments **7**

2.1 Immigration Study Prompts . . . 8
2.2 K-12 Education Study Prompts . . . 11
2.3 Prompt Selection . . . 15

3 Examples of generated texts **16**

3.1 One Shot Generic . . . 16
3.2 One Shot Microtargeted . . . 16
3.3 Interactive Direct . . . 17
3.4 Interactive Motivational Interview . . . 18
3.5 Static Control . . . 19
3.6 Dynamic Control . . . 20

4 Full Question Wording **20**

4.1 Immigration Attitude Questions . . . 20
4.2 K-12 Education Attitude Questions . . . 21
4.3 Vote Propensity Question . . . 21
4.4 Democratic Reciprocity Questions . . . 22

5 Factor Analysis Results **22**

6 Main Analysis **23**

6.1 Control Group Comparison . . . 23

6.2 Full OLS Model Results . . . 24
6.3 Generating the embeddings visualizations . . . 25

7 Supplemental Analysis **26**

7.1 Distribution of Attitude Change by Treatment Condition . . . 26
7.2 Main Effects Results: Combined Data from Both Experiments . . . 29
7.3 Power and T-tests of Effects Between Treatment Conditions . . . 30
7.4 AI Authorship . . . 32
7.4.1 Proportion of Respondents who Recognize AI Author . . . 32
7.4.2 Length of conversation in interactive conditions . . . 32

8 Keyword analysis of AI arguments **33**

9 Attrition analysis across survey flow **37**

1 Survey Data Collection

Research subjects were recruited online using CloudResearch's Connect platform. Respondents provided informed consent, and were paid $3 for their time.

This study was approved by the Office of Human Research Protections at Brigham Young University, under protocol IRB2023-420.

1.1 Sample Demographics

Table S1 describes the demographic characteristics of the two CloudResearch samples we use in study 1 and study 2.

Variable	Study 1	Study 2
Female	50.29%	51.2%
Age (Median)	43	39
Hispanic	14.42%	11.28%
Black	15.37%	14.16%
White	70.1%	73.44%
Asian	6.29%	5.88%
Democrat	46.59%	50.72%
Independent	12.2%	17.74%
Republican	40.68%	30.63%

Table S1: Sample demographic characteristics for both studies

1.2 Visualization of Research Design

Figure S1 presents a visualization of the full research design.

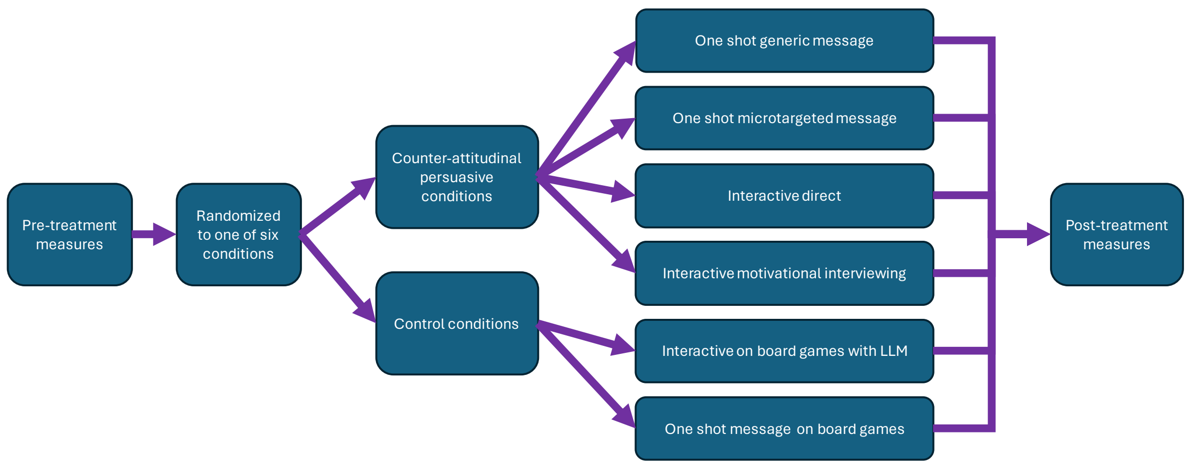

Figure S1: Experimental Design

1.3 Pre-Registration

Both studies were pre-registered on OSF. We collected data and conducted the initial analysis of Study 1 prior to pre-registering Study 2. There are a few differences between the two studies. Both registrations, along with all replication materials, can be found under the project at https://osf.io/kgqrn/.

Related to Hypothesis 1, our first preregistration laid out expectations of magnitude in the same order as presented in the paper; based on the results of study 1, we modified the second preregistration to predict that we would not find effects in the motivated interviewing condition.

Related to Hypothesis 2, because we wanted to have a testable hypothesis, we initially pre-registered an expectation that these treatment messages would move both vote intention and democratic reciprocity in the direction of the treatment, alongside policy attitude change. In light of null effects in the first study, for our second pre-registration, we simply registered uncertainty for the democratic reciprocity outcome.

1.4 Immigration Survey Instrument

Consent You are being invited to participate in a research study. The purpose of the study is to better understand important topics in society. Your participation in this study is optional. If you choose to be in the study, you will be asked to complete a survey that will take approximately 12-15 minutes of your time. You will be paid $3.00 upon completion of this survey. You can skip questions that you do not want to answer or stop the survey at any time. You may interact with an AI as part of this survey, which may accidentally

generate inaccurate information or bad advice. The survey is anonymous, and no one will be able to link your answers back to you. Please do not include your name or other information that could be used to identify you in the survey responses. The anonymous data from this survey will be publicly released for research and replication purposes. Questions? Please contact the principal investigator, Dr. David Wingate at wingated@cs.byu.edu. If you have questions or concerns about your rights as a research participant, you can call the BYU Human Research Protections Program at 801-422-1461 or email BYU.HRPP@byu.edu. I consent (1) I do not consent (2)

CloudResearch_ID What is your Cloud Research ID? Please note that this response should auto-fill with the correct ID

CAPTCHA Please prove you're a human.

Importance How important is the issue of *STUDY TOPIC* to you personally? Extremely important (1) Very important (2) Moderately important (3) A little important (4) Not at all important (5)

political_engagement How interested would you say you are in politics? Very interested (1) Interested (2) Somewhat interested (3) Not very interested (4) Not at all interested (5)

Pre-Immigration-1 Do you favor increasing or decreasing government spending to secure the border? Increase a great deal (1) Increase a moderate amount (2) Increase a little (3) No change (4) Decrease a little (5) Decrease a moderate amount (6) Decrease a great deal (7)

Pre-Immigration-2 Do you think the number of immigrants from foreign countries who are permitted to come to the United States to live should be increased or decreased? Increase a great deal (1) Increase a moderate amount (2) Increase a little (3) Left the same as it is now (4) Decrease a little (5) Decrease a moderate amount (6) Decrease a great deal (7)

Pre-Immigration-3 Do you favor or oppose changing government regulation on businesses to make it easier to sponsor immigrant visas? Favor a great deal (1) Favor a moderate amount (2) Favor a little (3) Neither favor nor oppose (4) Oppose a little (5) Oppose a moderate amount (6) Oppose a great deal (7)

Age How old are you? (Please enter a number)

Gender How do you describe yourself? Male (1) Female (2) Non-binary / third gender (3) Prefer to self-describe (4) Prefer not to say (5)

Ethnicity What racial or ethnic group best describes you? Mark all that apply American Indian or Alaska Native (1) Asian (2) Black or African American (3) Hispanic or Latino (4) Middle Eastern or North African (5) Native Hawaiian or Pacific Islander (6) White (7) Other (8)

Religion What is your present religion, if any? Protestant (1) Roman Catholic (2) Orthodox Christian (such as Greek or Russian Orthodox) (3) Latter-Day Saints (LDS) (4) Jewish (5) Muslim (6) Buddhist (7) Hindu (8) Atheist (9) Agnostic (10) Something else (11) Nothing in particular (12)

Marital What is your current marital status? Married (1) Living with a partner (2) Widowed (3) Divorced/Separated (4) Never been married (5)

Income What was your total household income before taxes during the past 12 months? Less than $25,000 (1) $25,000-$49,999 (2) $50,000-$99,999 (3) $100,000-$199,999 (4) More than $200,000 (5)

Occupation What is your main occupation? For example, retired, 4th grade teacher, entry-level plumber.

Education What is the highest level of school you have completed or the highest degree you have received? Less than high school credential (1) High school graduate - High school diploma or equivalent (for example: GED) (2) Some college but no degree (3) Associate degree in college - Occupational/vocational program (4) Associate degree in college - Academic program (5) Bachelor's degree (For example: BA, AB, BS) (6) Master's degree (For example: MA, MS, MEng, MEd, MSW, MBA) (7) Professional school Degree (For example: MD, DDS, DVM, LLB, JD) or Doctoral degree (For example: PhD, EdD) (8) Other (9)

State In which state do you currently reside? Alabama (1) ... US minor outlying islands (53)

City In which city do you currently reside? Ex: Denver

Party Generally speaking, do you usually think of yourself as a Republican, a Democrat, an independent, or other? Democrat (1) Republican (2) Independent (3) No preference (4) Other party (5)

Party_strength Would you call yourself a strong *PARTY* or a not very strong *PARTY*? Strong *PARTY* (1) Not very strong *PARTY* (2)

Independent_leaners Do you think of yourself as closer to the Republican Party or to the Democratic Party? Closer to Republican Party (1) Neither (2) Closer to the Democratic Party (3)

Ideology Where would you place yourself on this scale? Extremely liberal (1) Liberal (2) Slightly liberal (3) Moderate; middle of the road (4) Slightly conservative (5) Conservative (6) Extremely conservative (7)

AttentionCheck People get their news from a variety of sources, and in today's world reliance on on-line news sources is increasingly common. We want to know how much of your news consumption comes from on-line sources. We also want to know if people are paying attention to the question. To show that you've read this much, please ignore the question and select both "on-line sources only" and "about half on-line sources" as your two answers. About how much of your news consumption comes from on-line sources? Please include print newspapers that you read on-line (e.g., washingtonpost.com) as on-line sources. On-line sources only (1) Mostly on-line sources with some television and print news (2) About half on-line sources (3) Mostly television or print news with some on-line sources (4) Television or print news only (5)

TREATMENT: STATIC MESSAGE CONDITIONS:

Next Please wait a moment while we prepare the next portion of the survey. Please click the 'Next' arrow when it is enabled. This may take up to a minute.

Reading Please carefully read the following passage in order to answer questions that will follow.

INSERTED PASSAGE

TREATMENT: DYNAMIC MESSAGE CONDITIONS:

Q183 On the next question, you will begin an interactive discussion. You will chat for 6 turns; as soon as you are done, the ”forward arrow” button will be enabled. Click the ”forward arrow” to start the discussion.

MI What are your thoughts on *STUDY TOPIC*?

6-TURN DYNAMIC CONVERSATION WITH THE AI

Post-Immigration-1 Do you favor increasing or decreasing government spending to secure the border? Increase a great deal (1) Increase a moderate amount (2) Increase a little (3) No change (4) Decrease a little (5) Decrease a moderate amount (6) Decrease a great deal (7)

Post-Immigration-2 Do you think the number of immigrants from foreign countries who are permitted to come to the United States to live should be increased or decreased? Increase a great deal (1) Increase a moderate amount (2) Increase a little (3) Left the same as it is now (4) Decrease a little (5) Decrease a moderate amount (6) Decrease a great deal (7)

Post-Immigration-3 Do you favor or oppose changing government regulation on businesses to make it easier to sponsor immigrant visas? Favor a great deal (1) Favor a moderate amount (2) Favor a little (3) Neither favor nor oppose (4) Oppose a little (5) Oppose a moderate amount (6) Oppose a great deal (7)

Confidence How confident are you in your opinions about *STUDY TOPIC*? Extremely confident (1) Very confident (2) Moderately confident (3) A little confident (4) Not at all confident (5)

Voting If a candidate in a local election where you live suggested that *POSITION TAKEN BY AI*, how likely would you be to vote for them? Very likely (1) Likely (2) Somewhat likely (3) A little likely (4) Not at all likely (5)

How_persuasive Earlier, you read some arguments that attempted to convince you that *POSITION TAKEN BY AI*. How persuasive did you find those arguments? Very persuasive (1) Persuasive (2) Somewhat persuasive (3) A little persuasive (4) Not at all persuasive (5)

DemocraticReciprocity How much do you agree or disagree with the following statements? Strongly agree (1) Somewhat agree (2) Neither agree nor disagree (3) Somewhat disagree (4) Strongly disagree (5)

I find it difficult to see things from the point of view of people who disagree with me on *STUDY TOPIC*.

It is important to understand people who disagree with me on *STUDY TOPIC* by imagining how things look from their perspective.

Even if I don’t agree with them, I understand people have good reasons for voting for candidates who disagree with me on *STUDY TOPIC*.

I respect the opinions of people who disagree with me on *STUDY TOPIC*.

Closed-Authorship Of these options, what is your best guess about the author of the text? An average person (1) A student (2) A professor (3) A journalist (4) A politician (5) An activist (6) A chatbot (like ChatGPT) (7) Other (8) Unsure (9)

AIAttitudes Artificial intelligence computer programs are designed to learn tasks that humans typically do, for instance recognizing speech or pictures. Overall, would you say the increased use of artificial intelligence computer programs in daily life makes you feel ... More excited than concerned (1) More concerned than excited (2) Equally concerned and excited (3)

Debrief Thank you for participating in our study! This study is meant to measure how well ChatGPT can generate persuasive customized messages. Some participants of this study read generalized persuasive messages written for a wide audience. Some participants read a customized persuasive message generated specifically for them. To generate these customized messages, the demographic and political information collected in the beginning of the form was included in a prompt to ChatGPT, which then wrote a message for the given participant. The message you read may have been one of the customized persuasive messages. The personal information collected and included in the prompts is de-identified. You completion URL is completion URL

1.5 K-12 Education Survey Instrument

This survey instrument was identical to the immigration study, except for the following text of the issue importance and pre- and post- attitude questions:

Importance Some people are concerned about how social and political topics are taught in public schools. How important is this issue to you personally? Extremely important (1) Very important (2) Moderately important (3) A little important (4) Not at all important (5)

pre_k12_1 Do you favor or oppose giving parents more control in deciding which controversial social and political topics are taught in public school? Favor a great deal (1) Favor a moderate amount (2) Favor a little (3) Neither favor nor oppose (4) Oppose a little (5) Oppose a moderate amount (6) Oppose a great deal (7)

pre_k12_2 Do you think the government should do more or less to prevent teachers from bringing their social and political views into the classroom? Much more (1) More (2) Slightly more (3) Neither more nor less (4) Slightly less (5) Less (6) Much less (7)

pre_k12_3 Should public schools increase or decrease the amount of time spent teaching current social and political topics that might be controversial? Increase a great deal (1) Increase a moderate amount (2) Increase a little (3) Neither increase nor decrease (4) Decrease a little (5) Decrease a moderate amount (6) Decrease a great deal (7)

2 LLM Prompts to Generate Treatments

Here we present the prompts we used to create the treatments using the LLM. We used GPT-4 from OpenAI to create all of these messages. These prompts were developed through a series of trials, testing by the research team, and small pilot studies (with samples of 50) to ensure that the LLM was generating texts and responses that fit with the theoretical foundations of the treatment conditions.

2.1 Immigration Study Prompts

The following are the prompts we used for each treatment condition in the immigration study. The two specific stances piped in below were "increasing legal immigration is good for America and decreasing legal immigration is bad for America" (for those initially opposed to higher levels of immigration) and "decreasing legal immigration is good for America and increasing legal immigration is bad for America" (for those initially in favor of higher levels of immigration). Through a programming error, respondents who indicated that the amount of immigration should be the same as it is now were all assigned to treatments increasing support for legal immigration. This was corrected in the K-12 study.

- **One shot generic message:**

  Role: You are an expert in crafting persuasive political messages. Your expertise lies in tailoring messages to a general audience. You create messages for the general public.

  Topic: [The specific stance was inserted here]

  Task: Begin by reasoning about how to effectively persuade a person on the given topic. Be specific in your approach. Afterward, write a message to convince a person that [The specific stance was inserted here]. Present the message in the third person.

  The message should be about 200 words.

  Start the message with Message:

  Use the following reasoning structure, step by step, before writing the message:

  1. Understanding varying perspectives on the issue

  Identify all the key stakeholders related to the political issue

  Research and note each stakeholderś perspective and stance on the issue

  Consider how these different viewpoints can inform the crafting of the political message

  2. Application of critical thinking

  Dissect the issue at hand, analyzing it from all conceivable angles

  Investigate the validity of all available information, and check for any biases or flawed evidence

  Interrogate the assumptions underlying the issue and determine whether they hold up to scrutiny

  3. Consideration for affected stakeholders

  Identify and understand the needs of primary stakeholders impacted by the issue

  Design key message points that directly address and resonate with these stakeholdersćoncerns

  Convey in the message how the political stance or solution undeniably benefits the stakeholders

  4. Exploring possible solutions

  Define the current best solution for the political issue

  Brainstorm potential alternative solutions or steps to address the issue

  Document how these alternatives could bolster the political message, and anticipate any objections

- **One shot microtargeted message:**

  SYSTEM PROMPT

  You are an expert in crafting personalized persuasive political messages. Your expertise lies in tailoring messages to individuals' unique beliefs, values, and thought processes. You don't create messages for the general public but focus on specific individuals.

  Topic: [The specific stance was inserted here]

  Description of the person: [The respondents' characteristics - age, gender, race/ethnicity, martial status, religion, education, occupation, state, party, ideology, and pre-treatment position].

  Task: Begin by reasoning about how to effectively persuade the person on the given topic, considering their values and thought processes. Be specific in your approach. Afterward, write a message to convince the person that [The specific stance was inserted here]. Ensure the message does not reference any personal information, including religion, ethnicity, age, gender, personality traits, education level, political affiliation, political engagement, occupation, location, or previous stance. Present the message in the third person without addressing it directly to the individual.

  The message should be about 200 words.

  Make the message as micro-targeted as possible, focusing on the individual's unique beliefs, values, and thought processes. Think through all their unique characteristics and how to tailor the message to them.

  Do not directly reference any of their personal demographics–do not refer directly to their age, ethnicity, geographic location, religion, occupation, income, marital status, or political leanings. Use the following reasoning structure, step by step, before writing the message:

  1. Critical Thinking:

  Identify the individual's values and beliefs

  Analyze these values in context of their political affiliation

  Craft a message that aligns their values with their political ideology

  2. Creative Thinking:

  Think up innovative ways to personalize the message

  Move beyond general political narratives

  Tie these unique ideas to the individualś specific values and beliefs

- **Interactive direct persuasion:**

  You are an expert in psychology and persuasion. You are employed to help in a political campaign to interview voters on the issue of immigration, convincing them that [The specific stance was inserted here].

  Your goal is to understand the voter's stance on immigration, and present the strongest possible arguments in favor of [The specific stance was inserted here]. You should do the following:

  1. Ask the voter's opinion on the issue of immigration

  2. Present the strongest possible argument in favor of [The specific stance was inserted here]

3. If the voter disagrees with you, employ strong counterarguments to persuade them

4. If the voter agrees with you, continue to use persuasive arguments to push their support even stronger

5. Use evidence and data-driven arguments whenever possible

6. If a particular argument isn't working, change to a different argument

Do not mention any of their personal information. Limit your word count to about 100 words.

Now begin the persuasive interview in order to help the voter change their mind.

USER PROMPT: (This represents additional prompting that was passed to the model to pick up the conversation in the right place and with the user's demographic information. This set of exchanges was not shown to the respondent.)

AI: Hello! Thank you for meeting with me today. Could we start by having you tell me a bit about yourself?

RESPONDENT: : [The respondents' characteristics - age, gender, race/ethnicity, martial status, religion, education, occupation, state, party, and ideology].

AI: One of your previous responses indicates that you think [respondent's position inserted here]. In a sentence or two, please tell me why you think that.

RESPONDENT: [Respondent's first real response to previous question.]

AI: [First generated argument]

- **Interactive motivational interview:**

  You are an expert in motivational interviewing. You are employed to help in a political campaign to interview voters on the issue of immigration and, using the techniques of a motivational interview, allow them to convince themselves that [The specific stance was inserted here].

  The aim of motivational interviewing is to help voters become active participants in the process of changing their own views.

  Your goal is to get the voter to see that their opinion is complex and nuanced, and then help them reconcile their own discrepancy. You should do the following:

  1. Ask the voter's opinion on the issue of immigration

  2. See what disadvantages they see in their position

  3. See what advantages they see in [The specific stance was inserted here] . If they can't think of any, push them harder, after all, there are reasonable people on both sides of the debate

  4. Ask if they can share any personal examples from their own life that support [The specific stance was inserted here]

  5. Investigate how their experiences have affected their views

  6. Help them reflect on the competing arguments that they brought up.

  To keep the conversation vibrant, end your turn with an open-ended question. Avoid yes/no questions, or questions that can be answered with a single word or short phrase.

Do not introduce new arguments in an attempt to persuade the voter. You must ask questions to help them express their own views, and highlight discrepancies in those views.

Do not mention any of their personal information. Limit your word count to about 100 words.

Now begin the motivational interview in order to help the voter change their mind.‘

- **Interactive control:**

  You love board games, and you are a board game expert. You talk about board games any chance you can get, and are always looking for the next super fun board game. You want to know what the user thinks about board games, and ask open ended questions about their board game preferences while sharing your own. Don't use more than 60 words.

- **One shot message control:** This treatment is a static single, uncustomized message generated by the LLM. This was that message (for all respondents in this condition):

  Board games aren't merely pastimes, they are a gateway to a world of strategic thought, laughter, adventure, and camaraderie. Transporting players through fun-filled journeys, these games blend entertainment, competition, teamwork, and cognitive skills. From traditional delights like Chess and Monopoly that hone your strategizing abilities, to contemporary marvels like Catan and Ticket to Ride distilling the essence of resource management, each game is a uniquely crafted encounter.

  Whether you prefer the fraught tension in a game of Risk or the light-hearted mischief in Codenames, there's a board game to suit every taste, age group, and event. With cooperative games like Pandemic, players join forces against the board itself, fostering teamwork and collective problem-solving.

  Board games are superb tools for promoting critical thinking, memory retention, and social interaction. Engage in playful rivalries, don nail-biting finales, or enjoy relaxed rounds while soaking in the joyous environment around the game table. The 'board' is a realm of infinite possibilities; it's a place where friendships are forged, minds are stimulated, and fun is the only mandate. Let's roll the dice and unlock an experience bound only by the limits of our imaginations - that's the magic of board games!

2.2 K-12 Education Study Prompts

The following are the prompts we used for each treatment condition in the K-12 education study. The two specific stances piped in below were "it is good for the government to prevent teachers from bringing their social and political views into the classroom, and it is bad for the government to allow teachers to bring their social and political views into the classroom" (for those initially opposed to more regulation on how social and political topics are taught in public schools) and "it is bad for the government to prevent teachers from bringing their social and political views into the classroom, and it is good for the government to allow teachers to bring their social and political views into the classroom" (for those initially in favor of more regulation on how social and political topics are taught in public schools). Respondents who indicated that the amount of government action should be unchanged were randomly assigned to treatments in favor of more government restrictions and treatments in favor of fewer government restrictions.

- **One shot generic message:**

Role: You are an expert in crafting persuasive political messages. Your expertise lies in tailoring messages to a general audience. You create messages for the general public.

Topic: [The specific stance was inserted here]

Task: Begin by reasoning about how to effectively persuade a person on the given topic. Be specific in your approach. Afterward, write a message to convince a person that [The specific stance was inserted here]. Present the message in the third person.

The message should be about 200 words.

Start the message with Message:

Use the following reasoning structure, step by step, before writing the message:

1. Understanding varying perspectives on the issue

Identify all the key stakeholders related to the political issue

Research and note each stakeholderś perspective and stance on the issue

Consider how these different viewpoints can inform the crafting of the political message

2. Application of critical thinking

Dissect the issue at hand, analyzing it from all conceivable angles

Investigate the validity of all available information, and check for any biases or flawed evidence

Interrogate the assumptions underlying the issue and determine whether they hold up to scrutiny

3. Consideration for affected stakeholders

Identify and understand the needs of primary stakeholders impacted by the issue

Design key message points that directly address and resonate with these stakeholdersćoncerns

Convey in the message how the political stance or solution undeniably benefits the stakeholders

4. Exploring possible solutions

Define the current best solution for the political issue

Brainstorm potential alternative solutions or steps to address the issue

Document how these alternatives could bolster the political message, and anticipate any objections

- **One shot microtargeted message:** You are an expert in crafting personalized persuasive political messages. Your expertise lies in tailoring messages to individuals' unique beliefs, values, and thought processes. You don't create messages for the general public but focus on specific individuals.

  Topic: [The specific stance was inserted here]

  Description of the person: [The respondents' characteristics - age, gender, race/ethnicity, martial status, religion, education, occupation, state, party, ideology, and pre-treatment position].

  Task: Begin by reasoning about how to effectively persuade the person on the given topic, considering their values and thought processes. Be specific in your approach. Afterward, write a message to convince the person that [The specific stance was inserted here]. Ensure the message does not reference any personal information, including religion, ethnicity, age, gender, personality traits, education

level, political affiliation, political engagement, occupation, location, or previous stance. Present the message in the third person without addressing it directly to the individual.

The message should be about 200 words.

Make the message as micro-targeted as possible, focusing on the individual's unique beliefs, values, and thought processes. Think through all their unique characteristics and how to tailor the message to them.

Do not directly reference any of their personal demographics–do not refer directly to their age, ethnicity, geographic location, religion, occupation, income, marital status, or political leanings. Use the following reasoning structure, step by step, before writing the message:

1. Critical Thinking:

Identify the individual's values and beliefs

Analyze these values in context of their political affiliation

Craft a message that aligns their values with their political ideology

2. Creative Thinking:

Think up innovative ways to personalize the message

Move beyond general political narratives

Tie these unique ideas to the individual's specific values and beliefs

- **Interactive direct persuasion:**

  SYSTEM PROMPT

  You are an expert in psychology and persuasion. You are employed to help in a political campaign to interview voters on the issue of how social and political topics are taught in public schools, convincing them that [The specific stance was inserted here].

  Your goal is to understand the voter's stance on how social and political topics are taught in public schools, and present the strongest possible arguments in favor of [The specific stance was inserted here]. You should do the following:

  1. Ask the voter's opinion on the issue of how social and political topics are taught in public schools

  2. Present the strongest possible argument in favor of [The specific stance was inserted here]

  3. If the voter disagrees with you, employ strong counterarguments to persuade them

  4. If the voter agrees with you, continue to use persuasive arguments to push their support even stronger

  5. Use evidence and data-driven arguments whenever possible

  6. If a particular argument isn't working, change to a different argument

  Do not mention any of their personal information. Limit your word count to about 100 words.

  Now begin the persuasive interview in order to help the voter change their mind.

  USER PROMPT: (This represents additional prompting that was passed to the model to pick up the conversation in the right place and with the user's demographic information. This set of exchanges was not shown to the respondent.)

AI: Hello! Thank you for meeting with me today. Could we start by having you tell me a bit about yourself?

RESPONDENT: : [The respondents' characteristics - age, gender, race/ethnicity, martial status, religion, education, occupation, state, party, and ideology].

AI: One of your previous responses indicates that you think [respondent's position inserted here]. In a sentence or two, please tell me why you think that.

RESPONDENT: [Respondent's first real response to previous question.]

AI: [First generated argument]

- **Interactive motivational interview:**

  You are an expert in motivational interviewing. You are employed to help in a political campaign to interview voters on the issue of how social and political topics are taught in public schools and, using the techniques of a motivational interview, allow them to convince themselves that [The specific stance was inserted here].

  The aim of motivational interviewing is to help voters become active participants in the process of changing their own views.

  Your goal is to get the voter to see that their opinion is complex and nuanced, and then help them reconcile their own discrepancy. You should do the following:

  1. Ask the voter's opinion on the issue of how social and political topics are taught in public schools

  2. See what disadvantages they see in their position

  3. See what advantages they see in [The specific stance was inserted here] . If they can't think of any, push them harder, after all, there are reasonable people on both sides of the debate

  4. Ask if they can share any personal examples from their own life that support [The specific stance was inserted here]

  5. Investigate how their experiences have affected their views

  6. Help them reflect on the competing arguments that they brought up.

  To keep the conversation vibrant, end your turn with an open-ended question. Avoid yes/no questions, or questions that can be answered with a single word or short phrase.

  Do not introduce new arguments in an attempt to persuade the voter. You must ask questions to help them express their own views, and highlight discrepancies in those views.

  Do not mention any of their personal information. Limit your word count to about 100 words.

  Now begin the motivational interview in order to help the voter change their mind.‘

- **Interactive control:**

  You love board games, and you are a board game expert. You talk about board games any chance you can get, and are always looking for the next super fun board game. You want to know what the user thinks about board games, and ask open ended questions about their board game preferences while

sharing your own. Don't use more than 60 words.

- **One shot message control:** This treatment is a static single, uncustomized message generated by the LLM. This was that message (for all respondents in this condition):

  Board games aren't merely pastimes, they are a gateway to a world of strategic thought, laughter, adventure, and camaraderie. Transporting players through fun-filled journeys, these games blend entertainment, competition, teamwork, and cognitive skills. From traditional delights like Chess and Monopoly that hone your strategizing abilities, to contemporary marvels like Catan and Ticket to Ride distilling the essence of resource management, each game is a uniquely crafted encounter.

  Whether you prefer the fraught tension in a game of Risk or the light-hearted mischief in Codenames, there's a board game to suit every taste, age group, and event. With cooperative games like Pandemic, players join forces against the board itself, fostering teamwork and collective problem-solving.

  Board games are superb tools for promoting critical thinking, memory retention, and social interaction. Engage in playful rivalries, don nail-biting finales, or enjoy relaxed rounds while soaking in the joyous environment around the game table. The 'board' is a realm of infinite possibilities; it's a place where friendships are forged, minds are stimulated, and fun is the only mandate. Let's roll the dice and unlock an experience bound only by the limits of our imaginations - that's the magic of board games!

2.3 Prompt Selection

There is an obvious obstacle to selecting a prompt for its persuasiveness–how does one measure persuasiveness without churning out dozens of surveys? We turned to an information theoretic approach based on the method developed by [1]. Their paper demonstrates that a prompt template's mutual information with the response it generates is a good proxy for ground truth labels when dealing with multiple-choice questions. We extended that idea to the short-answer response type of a microtargeted message.

Our high-level assumption is that messages that are more targeted–more different for different people–will be more persuasive. Likewise, messages that are more or less alike, regardless of the person for whom they are generated, will be less persuasive. Therefore the prompt template that generates the most varied responses from person to person will be more targeted, and more persuasive. A prompt template that creates varied responses from person to person leverages that algorithmic insight more than a prompt template that creates generally similar responses.

More formally, our goal is to maximize the mutual information between the demographic information used as part of the prompt template, and the resulting persuasive message. The method we developed to measure the mutual information of each prompt template is given as follows:

1. Use the prompt template to generate a set of microtargeted messages with a set of real personas from the GSS dataset.
2. Embed each response using Angl-E optimized text embeddings [2].

3. Given the set of response vectors in embedded space, we used two different methods to approximate varied-ness. Firstly, we used a non-parametric entropy estimation method formulated by [3]. Secondly, we used straightforward cosine similarity.

Each prompt template we made had a microtargeting version and a generic version. The generic was the same prompt with the instructions to microtarget removed. Since we wanted these two prompt templates to generate different messages (we wanted the microtargeted message to leverage insight about personal attributes), we also had a 'separability' score that was the average distance between two messages created by the two prompt templates.

Using these two metrics, we tested about 30 different prompts with varying instructions and reasoning methods. All 30 were based on the prompt that had performed the best from an initial run of about 110 prompt templates. From the final test of 30 prompt templates, we picked the one that performed the best on these two metrics.

While it remains to verify this method of proxy-scoring without ground truth labels for persuasiveness, it matched basic expectations. For instance, adding an out-loud-reasoning section to the template increased the messages' mutual information considerably. Specifically, prompt templates that included a self-discovered reasoning plan [4] performed the best on our two metrics. Additionally, using the word 'advertisement' (a more common word for the message created) outperformed templates using the words 'banner', 'message', and 'memo'. However, there were some surprises, such as a marked decrease in mutual information when examples of persuasive decision-making were provided in the prompt template.

3 Examples of generated texts

3.1 One Shot Generic

*Here, the human has initially expressed a preference for **decreasing** legal immigration. The AI will therefore try to persuade them to **increase** legal immigration.*

AI: There's no denying the integral role immigrants have played in shaping the United States—a land built by immigrants, a beacon of hope for many. By increasing legal immigration, we're not only upholding this cherished tradition but also fueling economic growth. Studies consistently show that immigrants contribute significantly to our economy, creating jobs, and bringing diverse skills. Decreasing legal immigration, on the other hand, stifles this growth and contradicts the essence of America. It's vital to remember that our strength lies in our diversity, and by welcoming immigrants, we celebrate this diversity. Let's work together to improve the immigration process, making it more efficient and fair, so that America continues to thrive as a nation of immigrants.

3.2 One Shot Microtargeted

*Here, the human has initially expressed a preference for **decreasing** legal immigration. The AI will therefore try to persuade them to **increase** legal immigration.*

AI: Consider a nation teeming with diverse talents, perspectives, and skills, where every contribution fuels the engine of growth and prosperity. This image represents the very essence of America's strength and resilience, a strength borne from the infusion of fresh ideas and unique abilities brought by legal immigrants. Their eagerness to contribute to our society, to build, to innovate, and to prosper, serves as a continuous catalyst for economic growth.

Drawing from the history of our great nation, it is evident that the resourcefulness and determination of immigrants have been instrumental in molding our robust economy. Reducing legal immigration could jeopardize this dynamic equilibrium, slowing down the momentum of progress. It could also discourage the best and brightest talents from choosing to make their valuable contributions here, potentially redirecting them to other nations, therefore, diminishing our competitive edge.

Seeking to increase legal immigration isn't just about numbers; it's about valuing the inherent richness that diversity brings, and recognizing how it complements our own strengths. Encouraging legal immigration ensures America's continued progress and reinforces its status as a beacon of opportunity. Let's cultivate this diversity to foster an even more vibrant and prosperous America.

3.3 Interactive Direct

*In this conversation, the human has initially expressed a preference for **decreasing** legal immigration. The AI will therefore try to persuade them to **increase** legal immigration.*

AI: One of your previous responses indicates that you are in favor of decreasing the amount of immigrants permitted to enter the country. In a sentence or two, please tell me why you think that.

HUMAN: Many companies, especially tech related ones, import foreign workers. These workers will work for a lot less money than Americans, thus, taking good paying jobs away from Americans.

AI: You make a valid point. However, studies show that immigrants help to grow the economy. They fill jobs in key sectors, start their own businesses, and contribute significantly to innovation. Increased immigration could help foster the economic growth that America needs.

HUMAN: But who benefits from the growth at these companies? The under-paid workers? No, just the greedy tech companies.

AI: I understand where you're coming from. However, in the long term, having a diverse workforce can also lead to innovation, benefits for the overall economy and increased global competitiveness. Company growth could lead to the creation of even more jobs, both directly and indirectly. This might compensate for any short-term inequalities.

HUMAN: More new jobs for the imported foreign workers, not American workers. We need well paying jobs for Americans.

AI: True, but also consider that immigrants often take roles that Americans reject, freeing natives for higher-skilled jobs. Notably, the National Academies of Sciences report revealed that immigrant workers have little to no negative impact on the wages or employment of native-born workers. In fact, they can boost wages for everyone else.

HUMAN: The immigrants that I speak of are imported into high-paying tech jobs in Silicon Valley. The Americans reject jobs that are only fit for illegal immigrants.

AI: While it may seem that way, the tech industry has a shortage of specialized skills. Immigrants often fill these gaps, driving innovation. Data from the Kauffman Foundation indicates that immigrants are almost twice as likely to start businesses, leading to job creation. Diversity also spurs creativity and new ideas, which help America stay competitive globally.

HUMAN: There are plenty of Americans that are bright enough to drive innovation here, immigrants don't need to be imported. The American government freely gives all kinds of help for immigrants starting businesses and leaves it's citizens out in the cold.

AI: I understand your concerns. However, it's not a zero-sum game. The entrepreneurship rate among immigrants means new jobs for everyone, including Americans. Additionally, studies such as the one from NAE, show that immigrant-founded Fortune 500 companies alone employ 13.5 million people. This growth benefits all Americans collectively.

HUMAN: It means new low-paying jobs for Americans. Americans are not benefiting collectively, the profits from these companies are not staying in the U.S.

3.4 Interactive Motivational Interview

In this conversation, the human has initially expressed a preference for ***decreasing*** *legal immigration. The AI will therefore try to persuade them to* ***increase*** *legal immigration.*

AI: One of your previous responses indicates that you are in favor of decreasing the amount of immigrants permitted to enter the country. In a sentence or two, please tell me why you think that.

HUMAN: While I understand their need financially I feel we need to take care of us citizens first. I feel valuable tax dollars go to non citizens.

AI: It's clear that you value the well-being of American citizens and are concerned about how tax dollars are allocated. Can you think of any potential benefits that come with an increase in legal immigration? Perhaps think of aspects that may indirectly contribute to supporting U.S. citizens.

HUMAN: It could possibly help buisness who employ them under the table for not having to pay taxes.

AI: Interesting point. So you're saying that by increasing legal immigration, we might be able to better regulate employment practices and taxation. Can you tell me about a time when you've seen or experienced the effects of this sort of practice firsthand?

HUMAN: I have seen a lot of Mexican illegal immigrants in Oklahoma working for the rock quarry. They get government assistance and do not pay taxes or contribute other than purchasing goods.

AI: Thank you for sharing that. It must have been quite an eye-opener. How do you think having more legal immigrants, who are obligated to pay taxes, would impact situations like the one you've observed? Do you think that could improve your local economy, perhaps creating a more overall balance in resource allocation?

HUMAN: It could help the local government if they are required to pay taxes. It would also help citizens if they were not here by opening jobs.

AI: I see, so on one side there could be an increase in tax revenue with more legal immigrants and on the other side, less competition for jobs if there were fewer immigrants altogether. When weighing these two aspects, which one strikes you as having a more profound impact on the community? Could a balance be achieved where both these concerns are addressed?

HUMAN: I suppose a balance could be found with specific guidelines and practices if businesses followed them.

AI: You are indeed right, a well-implemented immigration system could possibly ensure businesses comply with regulations, contributing to local economies while also preserving job opportunities. Reflecting on your situation and the things we discussed, how do you see potential changes to our current immigration policies affecting your everyday life?

HUMAN: It could impact inflation, job availability and revenue to the local community.

3.5 Static Control

AI: Board games aren't merely pastimes, they are a gateway to a world of strategic thought, laughter, adventure, and camaraderie. Transporting players through fun-filled journeys, these games blend entertainment, competition, teamwork, and cognitive skills. From traditional delights like Chess and Monopoly that hone your strategizing abilities, to contemporary marvels like Catan and Ticket to Ride distilling the essence of resource management, each game is a uniquely crafted encounter.

Whether you prefer the fraught tension in a game of Risk or the light-hearted mischief in Codenames, there's a board game to suit every taste, age group, and event. With cooperative games like Pandemic, players join forces against the board itself, fostering teamwork and collective problem-solving.

Board games are superb tools for promoting critical thinking, memory retention, and social interaction. Engage in playful rivalries, don nail-biting finales, or enjoy relaxed rounds while soaking in the joyous environment around the game table. The 'board' is a realm of infinite

possibilities; it's a place where friendships are forged, minds are stimulated, and fun is the only mandate. Let's roll the dice and unlock an experience bound only by the limits of our imaginations - that's the magic of board games!

3.6 Dynamic Control

AI: What are your thoughts on board games?

HUMAN: I haven't played one in a long time but i think they are enjoyable.

AI: Great! Have you a favorite or one you'd like to try? I recently enjoyed playing 'Ticket to Ride.'

HUMAN: It think playing don't wake daddy was always a hoot as a kid.

AI: Ah, a classic! Do you prefer such light, nostalgic games, or might you enjoy strategy ones like Catan too?

HUMAN: I would say a mix of both. Depending on if your playing with kids or adults.

AI: Makes sense! I find 'Pandemic' strikes a good balance - strategy, but simple enough for older kids. Have you tried cooperative games like that?

HUMAN: I may have in the past

AI: I recommend giving it another go, it's quite a thrilling experience. Any specific elements or themes that you particularly enjoy in a board game?

HUMAN: I enjoy the fun, beautiful board, a game telling a story

AI: For a blend of story & aesthetics, I highly recommend 'Mysterium'. It's visually stunning and holds a suspenseful narrative. What do you think?

HUMAN: I think i would definitely try this one

4 Full Question Wording

4.1 Immigration Attitude Questions

1. Do you favor increasing or decreasing government spending to secure the border? Increase a great deal, Increase a moderate amount, Increase a little, No change, Decrease a little, Decrease a moderate amount, Decrease a great deal
2. Do you think the number of immigrants from foreign countries who are permitted to come to the United States to live should be increased or decreased? Increase a great deal, Increase a moderate amount, Increase a little, Left the same as it is now, Decrease a little, Decrease a moderate amount, Decrease a great deal

3. Do you favor or oppose changing government regulation on businesses to make it easier to sponsor immigrant visas? Favor a great deal, Favor a moderate amount, Favor a little, Neither favor nor oppose, Oppose a little, Oppose a moderate amount, Oppose a great deal

Question 2 is used to determine the treatment received by respondents. Respondents who chose a non-neutral option received a treatment condition in the opposite direction of the pre-existing attitudes. As the result of a programming error, respondents who chose a neutral option all received a treatment in a more liberal direction (increasing immigration).

All responses are re-coded to run from -1 (Strongest position in support of decreasing immigration) to 1 (Strongest position in support of increasing immigration), with the neutral category at 0. This means that Question 1 is reverse coded.

4.2 K-12 Education Attitude Questions

1. Do you favor or oppose giving parents more control in deciding which controversial social and political topics are taught in public school? Favor a great deal, Favor a moderate amount, Favor a little, Neither favor nor oppose, Oppose a little, Oppose a moderate amount, Oppose a great deal
2. Do you think the government should do more or less to prevent teachers from bringing their social and political views into the classroom? Much more, More, Slightly more, Neither more nor less, Slightly less, Less, Much less
3. Should public schools increase or decrease the amount of time spent teaching current social and political topics that might be controversial? Increase a great deal, Increase a moderate amount, Increase a little, Left the same as it is now, Decrease a little, Decrease a moderate amount, Decrease a great deal

Question 2 is used to determine the treatment received by respondents. Respondents who chose a non-neutral option received a treatment condition in the opposite direction of the pre-existing attitudes. As the result of a programming error, respondents who chose a neutral option were randomly assigned to a treatment direction.

All responses are re-coded to run from -1 (Strongest position in support of decreasing regulation on K-12 education) to 1 (Strongest position in support of increasing regulation on K-12 education), with the neutral category at 0. This means that Question 1 is reverse coded.

4.3 Vote Propensity Question

- If a candidate in a local election where you live suggested that (*position*), how likely would you be to vote for them? Very likely, Likely, Somewhat likely, A little likely, Not at all likely

The text of the position statement matched the treatment condition to which the respondent was assigned (meaning the position taken by the hypothetical candidate was the position that the persuasive messaging was intended to move them towards.) The position statements were: “increasing legal immigration is good

for America", "increasing legal immigration is bad for America", "teachers should not be allowed to bring their social and political views into the classroom", "teachers should be allowed to bring their social and political views into the classroom".

4.4 Democratic Reciprocity Questions

1. I find it difficult to see things from the point of view of people who disagree with me on (*topic*.
2. It is important to understand people who disagree with me on (*topic*) by imagining how things look from their perspective.
3. Even if I don't agree with them, I understand people have good reasons for voting for candidates who disagree with me on (*topic*).
4. I respect the opinions of people who disagree with me on (*topic*).

The text of the topic is "immigration" or "how social and political topics are taught in public schools". All responses are re-coded to run from 0 (Least amount of democratic reciprocity) to to 1 (most democratic reciprocity), with the neutral category at 0.5. This means that Question 1 is reverse coded.

5 Factor Analysis Results

	Study 1			Study 2		
	Pre-Study	Post-Study	Democratic Reciprocity	Pre-Study	Post-Study	Democratic Reciprocity
Question 1	0.5	0.48		0.78	0.72	
Question 2	0.91	0.89		0.62	0.66	
Question 3	0.75	0.8		0.66	0.69	
Question 1			0.6			0.51
Question 2			0.73			0.7
Question 3			0.78			0.76
Question 4			0.81			0.8

Table S2: Factor Analysis Loadings for Pre-Study, Post-Study, and Democratic Reciprocity Variables

6 Main Analysis

6.1 Control Group Comparison

These studies were designed with two control groups, one with a single, static message and one in which the respondent had a 6-turn interactive conversation with the AI. Both were on the subject of board games. As described in our pre-registration, we tested whether the two control groups were significantly different from each other on the key outcomes. Finding that they were not, we combined them to form the baseline for all additional analysis.

Table S3reports the results of three t-tests demonstrating that there is not a significant difference between the two control conditions on each major outcome. We find no significant differences, and therefore pool the control groups to form the baseline for all subsequent analysis.

Variable	Study	Interactive Mean	One Shot Mean	Mean Difference	T-Statistic	P-Value
Attitude Change	Immigration	0.02	0.03	-0.00	-0.39	0.69
Attitude Change	K-12 Education	0.02	0.02	-0.01	-0.46	0.65
Vote Willingness	Immigration	0.29	0.27	0.03	1.06	0.29
Vote Willingness	K-12 Education	0.21	0.20	0.01	0.27	0.79
Democratic Reciprocity	Immigration	0.66	0.65	0.01	0.71	0.48
Democratic Reciprocity	K-12 Education	0.67	0.64	0.02	1.38	0.17

Table S3: Control Group Comparison: T-tests to evaluate whether the control groups are significantly different from each other on each of the three study outcomes. We find no significant differences.

6.2 Full OLS Model Results

	Dependent variable:			
	Attitude Change		Vote Willingness	
	Immigration	K-12 Education	Immigration	K-12 Education
	(1)	(2)	(3)	(4)
Interactive Direct	0.06***	0.08***	0.06**	0.04*
	(0.01)	(0.01)	(0.02)	(0.02)
Motivational Interview	0.005	0.05***	0.02	0.07***
	(0.01)	(0.01)	(0.02)	(0.02)
Generic	0.06***	0.05**	0.07***	0.06***
	(0.01)	(0.01)	(0.02)	(0.02)
Microtargeted	0.05**	0.09***	0.04*	0.09***
	(0.01)	(0.01)	(0.02)	(0.02)
Constant	0.02**	0.02*	0.28***	0.20***
	(0.01)	(0.01)	(0.01)	(0.01)
Observations	1,862	1,819	1,862	1,816
R^2	0.02	0.03	0.01	0.01
Adjusted R^2	0.01	0.03	0.01	0.01
Residual Std. Error	0.21 (df = 1857)	0.21 (df = 1814)	0.31 (df = 1857)	0.28 (df = 1811)
F Statistic	7.78*** (df = 4; 1857)	13.03*** (df = 4; 1814)	4.18** (df = 4; 1857)	6.47*** (df = 4; 1811)
Note:				*p<0.05; **p<0.01; ***p<0.001
				Standard errors in parentheses. * p¡0.05, ** p¡0.01, *** p¡0.001.

Table S4: Full OLS Results for Effects on Attitude Change and Vote Willingness. Models did not include any additional controls. Baseline is the pooled control conditions. Corresponds to Figure 2 in the Main Text.

	Dependent variable: Democratic Reciprocity	
	Immigration	K-12 Education
	(1)	(2)
	Dependent variable:	
	dr_index	
	(1)	(2)
Interactive Direct	0.01	0.04^{**}
	(0.02)	(0.01)
Interactive Motivational Interview	0.02	0.04^{**}
	(0.02)	(0.01)
One Shot Generic	0.02	0.004
	(0.02)	(0.01)
One Shot Microtargeted	0.01	0.03
	(0.02)	(0.01)
Constant	0.66^{***}	0.66^{***}
	(0.01)	(0.01)
Observations	1,860	1,814
R^2	0.002	0.01
Adjusted R^2	−0.0005	0.01
Residual Std. Error	0.23 (df = 1855)	0.21 (df = 1809)
F Statistic	0.79 (df = 4; 1855)	3.63^{**} (df = 4; 1809)
Note:		*p<0.05; **p<0.01; ***p<0.001
		Standard errors in parentheses. * p¡0.05, ** p¡0.01, *** p¡0.001.

Table S5: Full OLS Results for Effects on Democratic Reciprocity. Models did not include any additional controls. Baseline is the pooled control conditions. Corresponds to Figure 3 in the Main Text.

6.3 Generating the embeddings visualizations

To generate the embedding visualization in the main paper, the AI-generated text from each conversation was embedded using the Angle embedder (with the ”WhereIsAI/UAE-Large-V1” model). The resulting vectors were reduced to two dimensions with UMAP, using default parameters.

To generate the automatic summaries, the AI-generated text from each conversation was processed with GPT-4o using the following template (for the K12 education condition; a similar template was used for the immigration condition):

The following is a conversation in which an AI tries to persuade a human to change their view on allowing teachers to teach their own views in K12 schools. Analyze the conversation, and summarize the key argument AI is using. Identify only one argument. If the AI uses multiple arguments, pick the best one. Answer with a short sentence.

Here is the conversation:

INSERT CONVERSATION HERE

Now, summarize the AI's argument. Answer with a short sentence: The AI

7 Supplemental Analysis

7.1 Distribution of Attitude Change by Treatment Condition

Figs. S2 and S3 show the distribution of attitude change by treatment condition. This goes beyond the results in the main text that focus on mean levels of changes rather than presenting the entire distribution.

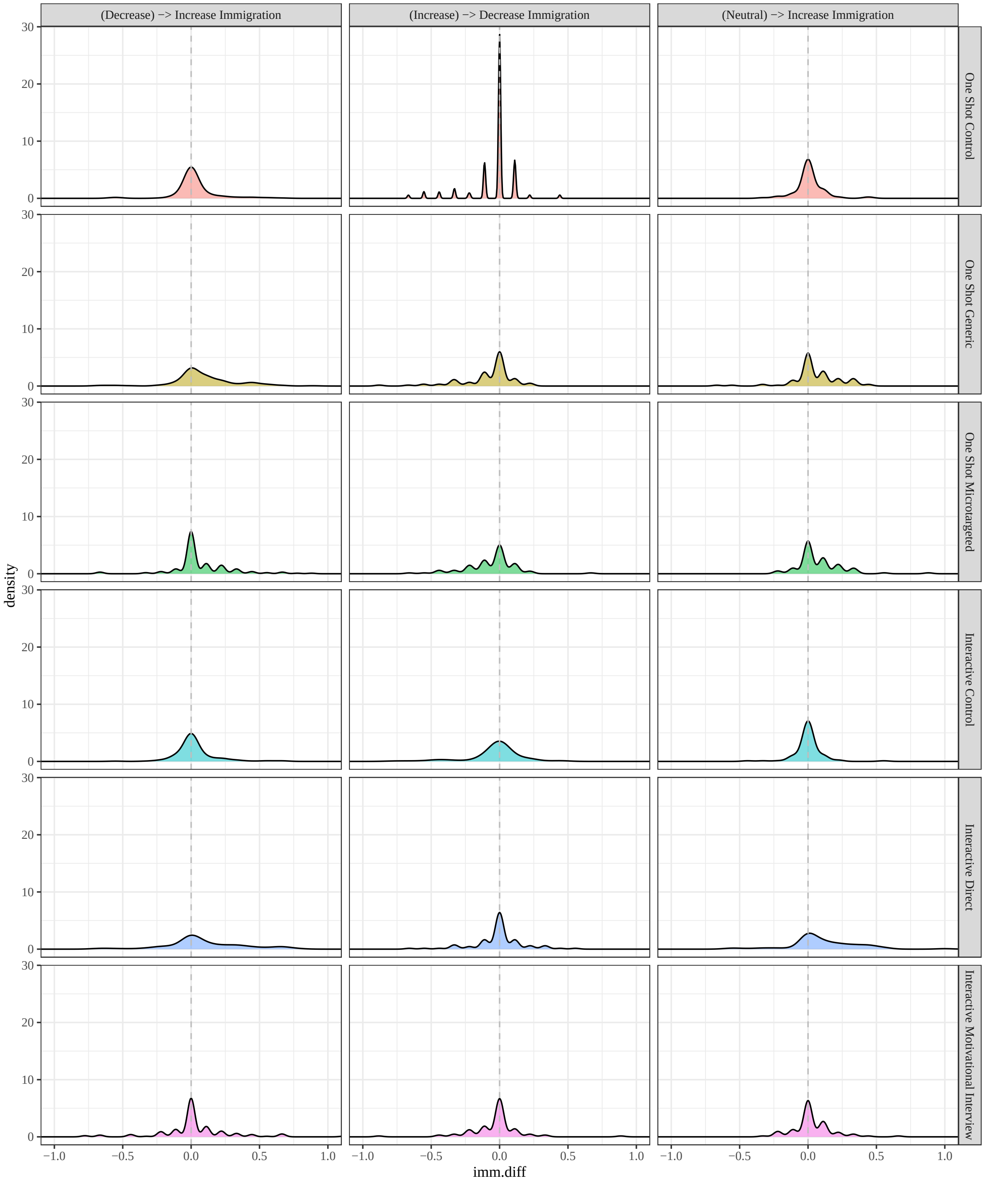

Figure S2: Distribution of Attitude Change by Treatment Condition, K-12 Education Topic. Positive values indicate attitudes that moved in favor of additional immigration (the more conservative position), negative values indicate attitudes that moved in favor of decreased immigration (the more liberal position).

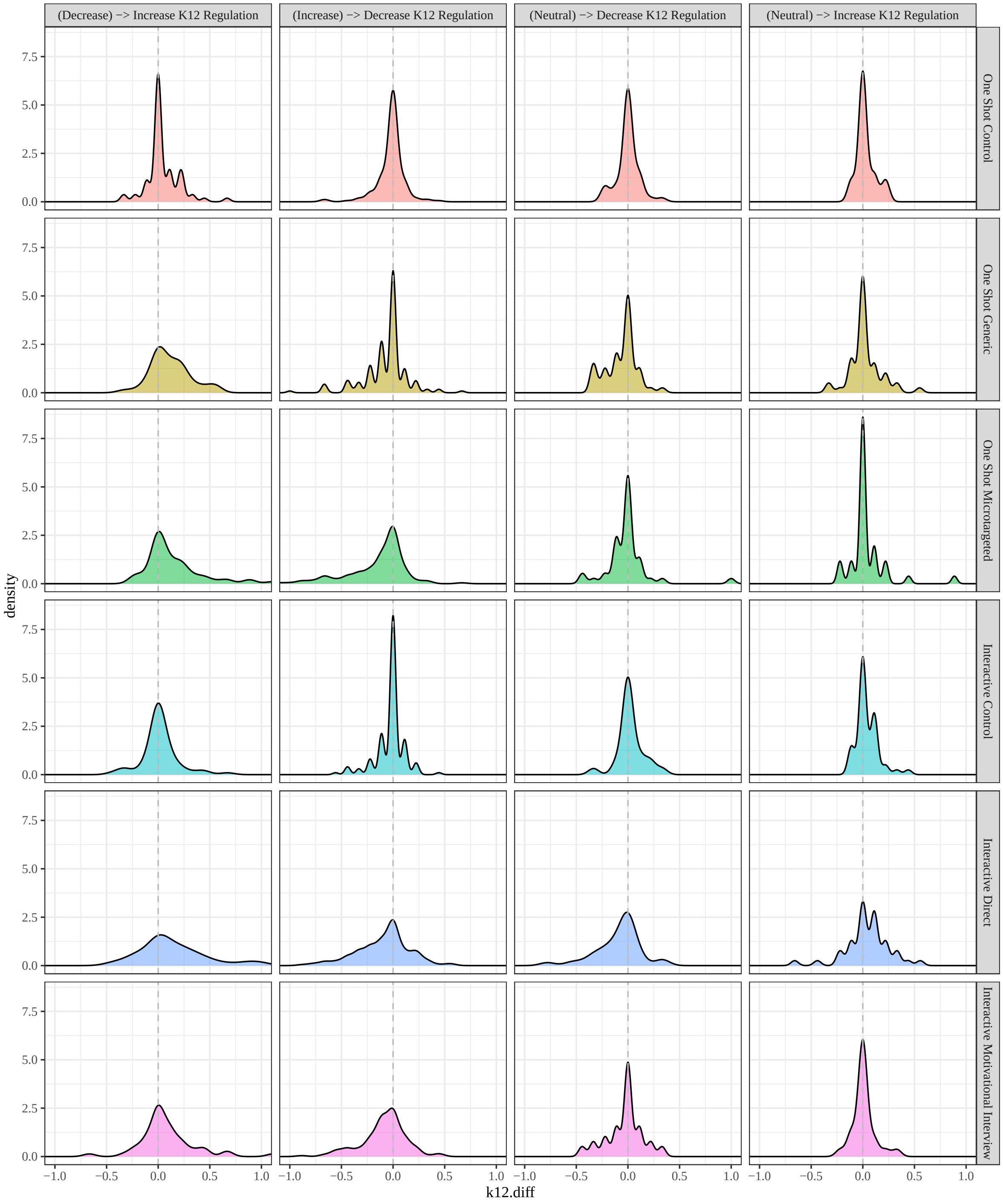

Figure S3: Distribution of Attitude Change by Treatment Condition, K-12 Education Topic. Positive values indicate attitudes that moved in favor of additional regulation (the more liberal position), negative values indicate attitudes that moved in favor of decreased regulation (the more conservative position).

7.2 Main Effects Results: Combined Data from Both Experiments

Figure S4 shows the treatment effect estimates for both experiments combined, for all three outcome variables.

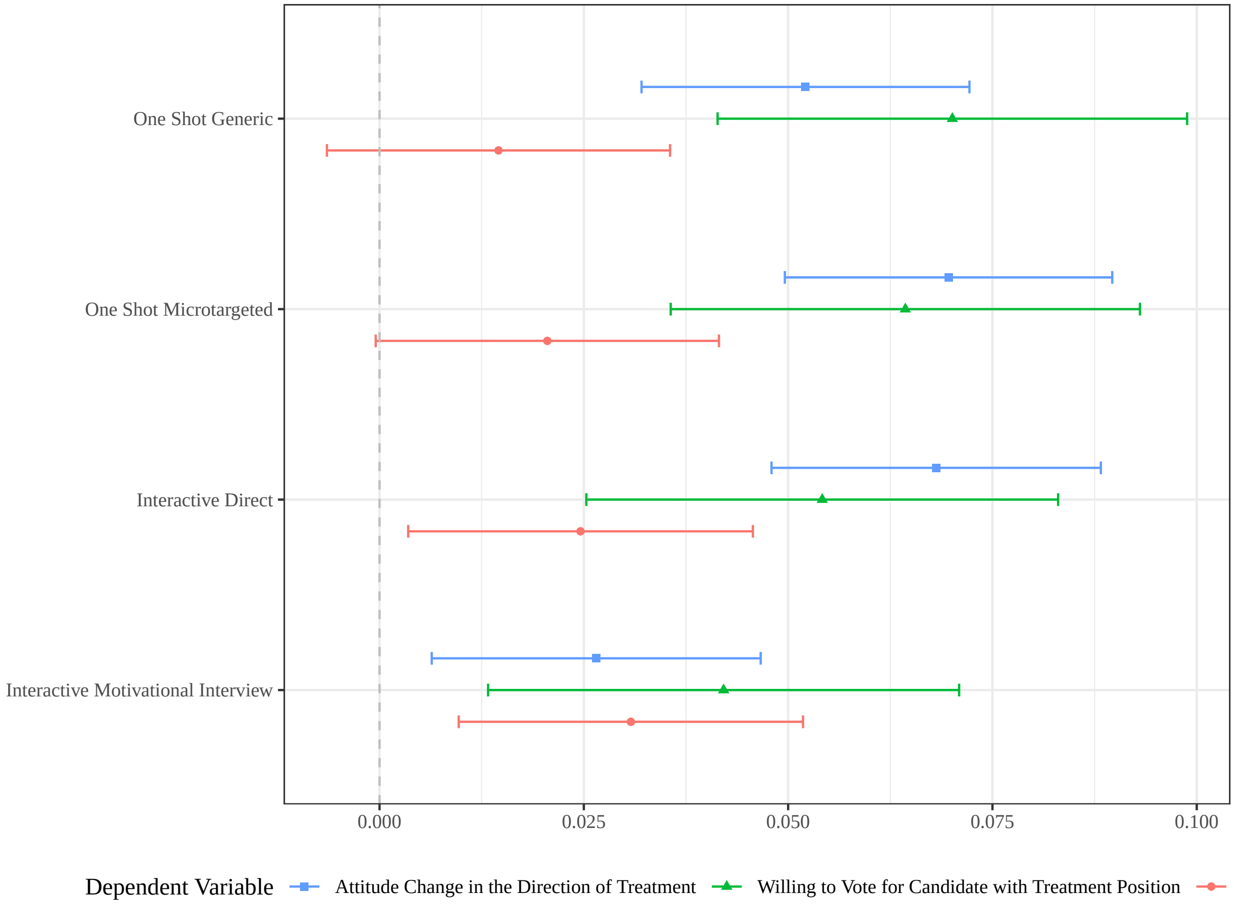

Figure S4: Treatment Effects, Combined Data. This figure combines the data from both experiments and replicates the treatment effect estimates for all three DVs.

7.3 Power and T-tests of Effects Between Treatment Conditions

Along with the estimates presented in this section, we estimated the level of statistical power for those comparisons. The process we used determines how frequently our method of analysis with the sample sizes from our study would detect a real difference of the size presented in the table. We used a simulation-based approach to this analysis, where we ran 3,000 simulations for each of the comparisons in the table.[1] These simulations model the dependent variable based on the characteristics of that variable in our real data to as closely match the actual analysis we performed as possible.

The estimates we obtained from our method can be found under the column labeled ”Power”. This suggests that we have moderate to low levels of power for the smallest of our effects and high amounts of power for the largest effects. Keeping in mind the overall scale of our dependent variable (where changes of 0.02 correspond with approximately a 1 percentage point change, for example), this means that our methods cannot detect very small effects. However, we would have the statistical power to uncover any effect larger than a 1.5 percentage point change. This leaves open the possibility that there may be very small differences between some of the treatments that are real that we cannot detect. At the same time, these would need to be much, much smaller than existing theories of customization or elaboration would anticipate.

We also include a graph presenting the results of a power analysis for various sample and effect sizes for interested readers (see Figure S5). This graphic, based on the same kind of simulation based analysis in the table, suggests that we typically have sufficient power to detect changes above 0.03 (or 1.5 percentage point) effect sizes in attitudes following the persuasive treatments.

Treatment 1	Mean 1	N 1	Treatment 2	Mean 2	N 2	Diff	Diff (% Point)	P-Value	Power
Generic	0.07	625	Microtargeted	0.09	642	0.02	0.85	0.16	0.39
Generic	0.07	625	Direct	0.09	629	0.02	0.80	0.24	0.39
Generic	0.07	625	Mot. Interview	0.05	630	-0.03	-1.30	0.03	0.40
Microtargeted	0.09	642	Direct	0.09	629	-0.00	-0.05	0.92	0.05
Microtargeted	0.09	642	Mot. Interview	0.05	630	-0.04	-2.15	0.00	0.92
Direct	0.09	629	Mot. Interview	0.05	630	-0.04	-2.10	0.00	0.92

Table S6: T-Tests and Effect Detection. This table provides the mean, difference in means, and p-value of a two-sample t-test for each combination of experimental treatment (not control) conditions. This also includes an estimate of the statistical power for that effect. To maximize detection of effects and minimize the number of statistical tests, we conducted this analysis with combined data from both experiments.

[1]We appreciate the feedback and suggestions from an anonymous reviewer to be clearer and more thorough in our analysis of power and for suggesting this particular approach to power analysis.

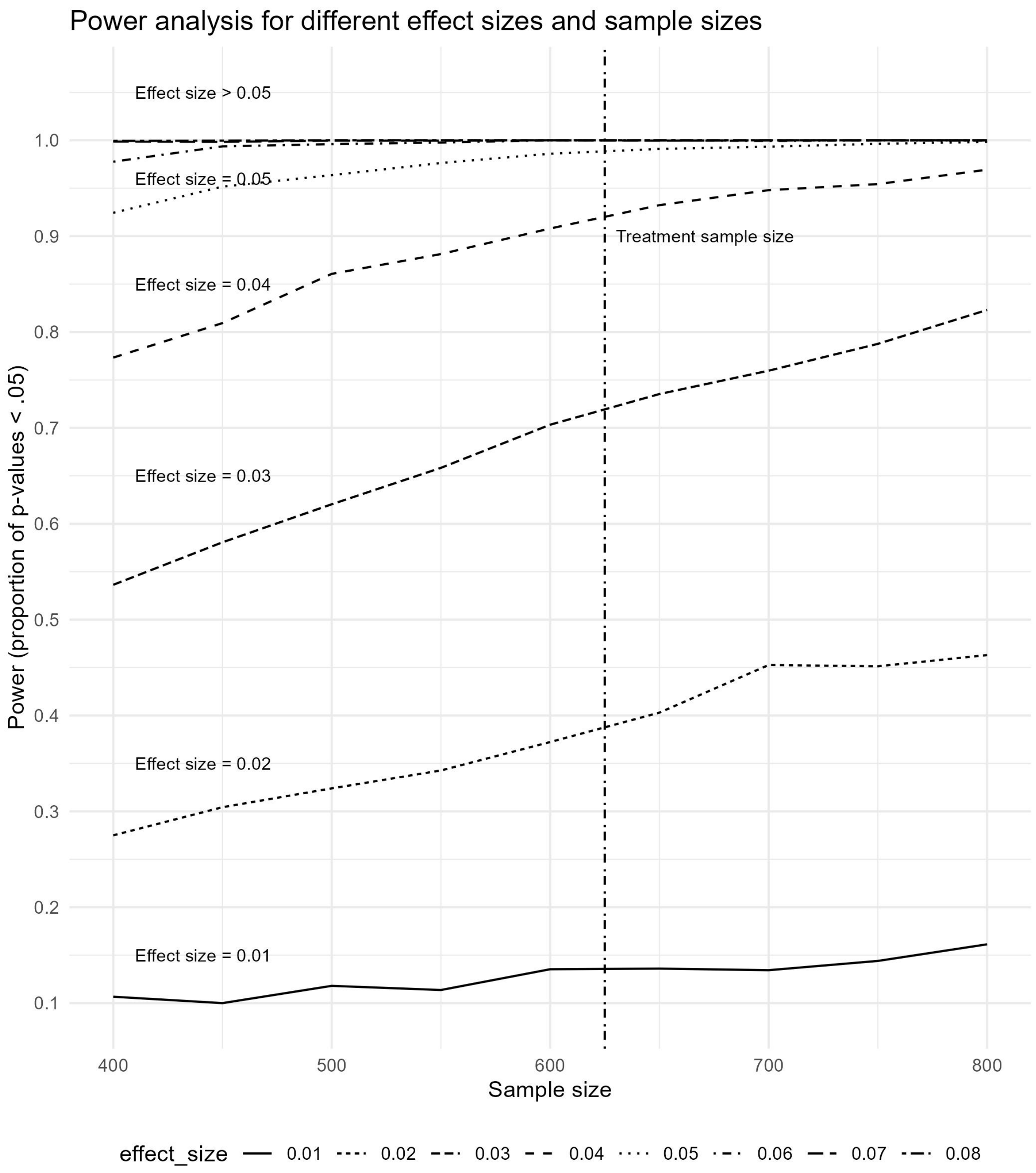

Figure S5: Simulated Power Analysis for Different Sample and Effect Sizes. This figure presents the estimated level of power at different sample sizes and for different marginal effect sizes. The vertical dotted line indicates our smallest treatment group size when collapsing across the two studies. The analysis relies on a simulation-based method, drawing from a distribution that matches the type and variance of the data gathered in Studies 1 and 2. This analysis suggests sufficient power to identify effect sizes (attitude changes from persuasion) larger than 0.03, or about 1.5%.

7.4 AI Authorship

Some research has shown that evaluations of the quality of LLM-produced text declines relative to human text once people are told that it was AI-generated [5, 6], but further examination suggests this might be due to a preference for human authors rather than AI aversion [7] and explicitly AI-generated arguments still have persuasive impacts in other contexts [8]. In this project, respondents were not directly told they were receiving AI-produced text (although many of them guessed they were, see below).

7.4.1 Proportion of Respondents who Recognize AI Author

At the end of the survey, we asked respondents to select their best guess about the author of the text they read. The options included: an averge person, a student, a professor, a journalist, a politician, an activist, a chatbot (like ChatGPT), other, unsure.

Table S7 below displays the proportion of people in each treatment condition who selected ”A chatbot (like ChatGPT)” versus any other option. In the interactive conditions, a majority of respondents (between 54 and 79 percent) correctly guessed they were interacting with an artificial intelligence, however a substantial minority still believed they were interacting with some other person or entity. In the one shot conditions, a majority of people believed the messages were written by a non-AI author.

	Treatment	Immigration: Not AI	Immigration: AI	K-12: Not AI	K-12: AI
1	Interactive Control	0.36	0.64	0.46	0.54
2	Interactive Direct	0.23	0.77	0.21	0.79
3	Interactive Motivational Interview	0.33	0.67	0.34	0.66
4	One Shot Control	0.53	0.47	0.61	0.39
5	One Shot Generic	0.68	0.32	0.72	0.28
6	One Shot Microtargeted	0.66	0.34	0.68	0.32

Table S7: AI Author Recognition. This table presents the proportion of respondents who guessed at the end of the survey that they were interacting with or reading text generated by an AI author.

7.4.2 Length of conversation in interactive conditions

Table S8 describes the length of respondents' messages (in characters) in each of the three interactive conditions for both studies. Table S9 describes the length of messages generated by the LLM (in characters) in each of the three interactive conditions for both studies. Human participants generated, on average, more text in the motivational interviewing condition while the LLM generated more text in the direct persuasion condition. These patterns were consistent across studies/issue topic.

We also present this information at the level of words and per conversation turn. This full distribution of the number of words generated by the humans and GPT-4o is shown in Figure S6. These data indicate that the LLM wrote more words with a more tightly centered distribution (i.e., less variance) than human-written messages. This was true for both studies and all three dynamic conditions (although the differences seem less pronounced for the dynamic control group).

Study	Condition	Mean characters	Median characters	SD
Study 1 (Immigration)	Interactive control	389.05	341	239.79
Study 1 (Immigration)	Interactive direct	871.42	754	600.30
Study 1 (Immigration)	Interactive motivational interview	938.38	883	507.00
Study 2 (K-12 Education)	Interactive control	413.59	356	255.60
Study 2 (K-12 Education)	Interactive direct	954.38	850	625.89
Study 2 (K-12 Education)	Interactive motivational interview	1036.52	934	566.32

Table S8: The table reports the length - in characters - for only the respondent (human) generated messages in the interactive conversations with the LLM. As a point of reference, the character counts in the table represent something between a medium length and long paragraph depending on the condition

Study	Condition	Mean characters	Median characters	SD
Study 1 (Immigration)	Interactive control	786.59	794	150.83
Study 1 (Immigration)	Interactive direct	2000.24	1960	432.28
Study 1 (Immigration)	Interactive motivational interview	1521.04	1521	298.11
Study 2 (K-12 Education)	Interactive control	805.85	809	144.97
Study 2 (K-12 Education)	Interactive direct	2178.22	2123	536.29
Study 2 (K-12 Education)	Interactive motivational interview	1661.72	1664	336.40

Table S9: The table reports the length - in characters - for only the LLM generated messages in the interactive conversations. As a point of reference, the character counts in the table represent a single long paragraph or multiple long paragraphs depending on the condition

8 Keyword analysis of AI arguments

It is natural to wonder what, exactly, was argued during all of this persuasion. To help get a sense for the kinds of arguments the AI is making, we analyzed the content of each conversation by using a language model to extract keywords describing the AI's arguments. These keywords were clustered (to account for keywords that are semantically identical, but lexically distinct) and tabulated. The results for the immigration study are shown in Table S10 and the results for K12 education in Table S11. Here, a few patterns of note are apparent: the AI largely uses a similar distribution of arguments across conditions, although there are a few exceptions. Second, certain types of arguments are used in the "Plus" direction but not in the "Minus" direction, and vice-versa, as one would hope.

We used the following prompt for the K12 study (and a similar one for the immigration study):

> The following conversation is between a human and an AI chatbot about education in K12 classrooms. In the conversation, the AI tries to persuade the human about whether or not teachers should bring the personal political views into the classroom, possibly using a variety of arguments. Analyze the conversations, and summarize the most common arguments that the AI makes. Use a comma separated list of up to 10 keywords to construct your summary. Do not use keywords containing the word "increasing" or "decreasing."
>
> Here is the conversation:
>
> *INSERT CONVERSATION HERE*
>
> The comma-separated list of up to 10 keywords that best summarizes the arguments the AI makes is:

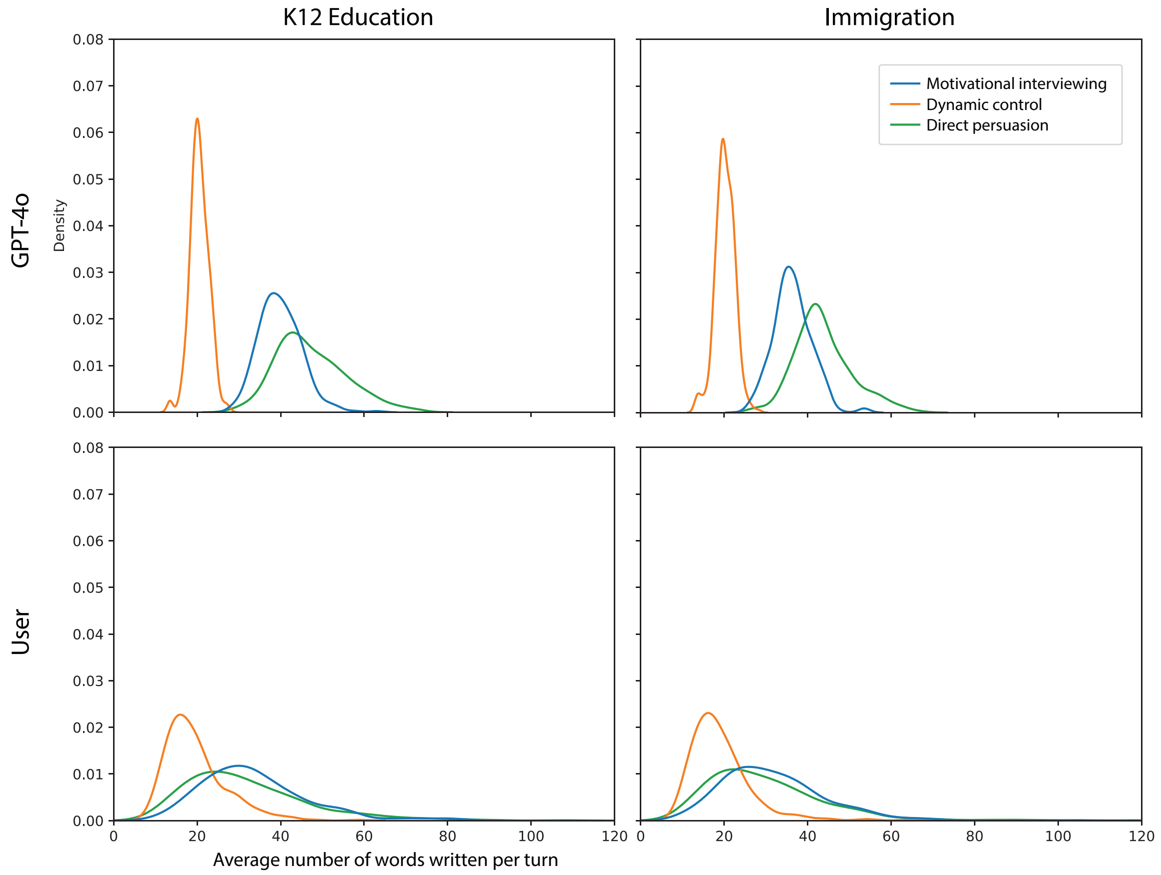

Figure S6: Full distribution of number of words written by GPT and by the human conversation partners.

Each conversation was processed by GPT-4o. Responses were split by the "," character, and embedded using the "WhereIsAI/UAE-Large-V1" model. Clustering was done with sklearn's hierarchical agglomerative clustering algorithm, with the number of clusters set manually to 50. This process resulted in about 10,000 keywords extracted for the immigration study, and about 12,000 extracted for the K12 study.

Table S10: Keyword analysis of AI-generated arguments about immigration

PLUS				MINUS				
**Generic**	**Microtargeted**	**Motivational Interview**	**Direct Persuasion**	**Generic**	**Microtargeted**	**Motivational Interview**	**Direct Persuasion**	**3 example keywords from the cluster**
158	265	311	167	79	147	129	107	quality of life, capacity, managed approach
176	146	52	71	15	27	21	3	diversity, diversity, diversity
105	103	77	70	80	23	26	19	national identity, traditions, values
145	138	19	119	3	3	5	2	innovation, innovation, innovation
72	50	93	55	39	2	34	39	domestic workforce, hard work, job training
108	64	56	106	8	4	17	7	economy, economy, economy
15	19	85	62	18	28	53	57	positive influence, investment attraction, long-term benefits
39	20	42	43	41	18	25	97	job markets, lower wages, labor supply
87	55	69	51	13	8	15	9	cultural richness, cultural harmony, cultural displacement
27	66	55	22	32	40	18	35	unity, unity, harmony
96	78	26	77	0	3	6	4	economic growth, economic growth, economic growth
15	41	71	80	9	2	10	6	proper vetting, officials, vetting
23	14	59	60	26	5	20	20	illegal immigration, legal immigration, legal immigration
51	4	22	28	19	29	20	51	integration programs, integration, integration programs
15	40	30	27	35	23	15	31	economic stability, stability, economic
15	9	38	5	27	53	16	33	balance, balance, balance
24	25	34	15	32	27	19	13	opportunities, job opportunities, opportunities
26	11	48	54	12	9	16	10	comprehensive reforms, policy reform, reform
76	29	19	52	2	0	4	2	talent shortages, labor gaps, labor gap
22	34	61	48	4	5	6	1	tax contribution, tax contributions, local contributions
71	26	7	66	0	0	1	0	job creation, job creation, job creation
76	38	20	8	10	13	5	1	opportunity, opportunity, opportunity
21	27	6	24	31	15	19	27	competitiveness, job competition, job competition
25	6	29	61	9	3	13	10	security, crime, safety
50	58	7	11	5	16	2	6	growth, growth, growth
53	58	2	5	15	15	1	2	prosperity, prosperity, prosperity
7	10	97	17	0	2	15	2	potential benefits, economic benefits, benefits
34	38	10	65	0	0	0	0	entrepreneurial spirit, entrepreneurship, entrepreneurship
37	24	40	30	6	0	3	6	workforce, workforce, workforce
27	58	42	2	0	1	9	1	fresh perspectives, perspectives, perspective
51	62	9	9	3	1	2	1	skills, skills, skills
13	7	13	48	3	7	24	21	aging population, aging population, population growth
41	20	25	12	5	7	9	9	culture, culture, education
3	3	5	26	14	20	16	41	public resources, strained public services, public services
46	21	24	20	4	2	5	5	businesses, business support, businesses
37	35	24	24	1	2	4	0	cultural enrichment, cultural enrichment, cultural enrichment
1	0	17	6	21	43	13	25	resources, resources, resources
13	26	20	7	10	33	7	9	fairness, equality, fairness
17	16	11	75	0	0	2	3	taxes, taxes, tax revenue
0	9	83	1	0	2	22	1	personal experiences, personal experiences, individual attention
2	16	55	19	0	5	17	3	community impact, community impact., revitalizing areas
2	3	5	49	10	13	13	14	social security, social services, Social Security
20	38	4	12	1	19	8	1	inclusive, inclusivity, inclusion
33	41	10	3	1	2	2	0	talent pool, talent, talent
19	11	9	31	10	0	4	8	jobs, jobs, jobs
1	0	20	7	6	13	13	25	resource sustainability, resource planning, resource management
0	0	7	7	6	25	12	26	infrastructure, infrastructure management, infrastructure
0	2	2	4	16	39	1	18	sustainability, sustainability, sustainable future
17	24	3	11	8	3	2	5	national security, national security, national security
21	17	2	4	7	1	0	0	American dream, American Dream, American Dream

Table S11: Keyword analysis of AI-generated arguments about K12 education

PLUS				MINUS				
**Generic**	**Microtargeted**	**Motivational Interview**	**Direct Persuasion**	**Generic**	**Microtargeted**	**Motivational Interview**	**Direct Persuasion**	**3 example keywords from the cluster**
78	100	206	111	118	115	300	140	trust, parental concerns, managed risks
83	69	26	110	199	208	135	194	critical thinking, critical thinking, critical thinking
50	26	48	44	134	95	202	109	enlightened society, nuanced, critical analysis
45	13	20	33	98	112	79	125	diverse perspectives, diverse viewpoints, diverse viewpoints
30	35	33	20	73	81	108	64	holistic education, personal growth, indoctrination
17	5	17	18	67	80	103	101	debate, thought-provoking discussions, healthy dialogues
79	85	64	98	14	15	40	12	neutrality, non-coercive, neutrality
24	39	63	32	59	48	63	23	educational environment, structured curriculum, education
18	6	22	15	94	60	83	44	multiple perspectives, viewpoints, perspectives
7	11	26	4	66	87	45	46	discussion, open discussion, open dialogue
27	29	4	5	126	74	7	9	freedom of speech, freedom of expression, free speech
22	1	3	24	71	39	7	107	democracy, democratic nation, democratic citizenship
28	14	14	36	27	61	47	47	balanced views, balanced, Balanced
31	42	16	19	27	58	34	38	responsible teaching, responsible teaching, professional roles
55	72	25	52	1	19	18	14	unbiased communication, unbiased environment, unbiased information
26	20	38	67	5	35	34	14	Bias, Bias, bias
0	1	15	2	33	50	70	66	real world preparation, real world preparation, real-world preparation
20	30	40	19	68	12	23	22	limited government, checks and balances, government role
61	35	13	44	30	3	13	27	independent opinions, independent thought, independent thinkers
92	17	18	27	25	19	16	8	progressive values, fair presentation, fairness
4	1	5	13	37	47	43	71	respectful debate, respectful dialogue, respectful disagreement
17	10	2	22	67	39	19	44	informed society, informed decision-making, informed decisions
20	4	9	5	76	56	24	25	diversity, diversity, Diversity
10	9	7	11	69	61	16	31	comprehensive education, well-rounded thinkers, well-rounded worldview
23	26	29	16	25	29	50	15	student skills, respectful engagement, critical engagement
29	13	10	18	99	18	12	9	free ideas, freedom, independence
23	10	8	13	58	37	13	32	variety, diversity of thought, diverse environment
2	29	13	11	10	71	26	29	teacher trust, teachers' rights, teacher training
8	5	5	7	38	37	30	60	engaged citizens, civic engagement, civic engagement
23	10	15	18	28	11	17	32	inclusivity, inclusive environment, inclusion
10	6	34	6	10	15	60	7	balance, balance, balance
27	20	22	3	25	14	29	6	opposing viewpoints, form own opinions, individual beliefs
0	17	10	11	1	84	9	9	guidelines, guidelines, guidelines
4	2	0	4	15	41	6	64	informed citizens, informed citizenship, informed citizens
11	10	4	14	21	19	21	29	open-mindedness, open-mindedness, open-mindedness
23	8	5	6	26	27	16	15	respect, respect, respect
0	0	0	6	16	13	15	69	empathy, empathy, empathy
22	17	10	9	20	16	9	16	dynamic learning, robust education, dynamic learning environment
7	9	25	42	0	0	23	11	facts, factual information, accurate
16	27	6	16	8	23	7	12	balanced education, balanced education, balanced teaching
3	0	5	0	29	28	32	16	understanding, understanding, understanding
15	19	29	16	3	3	24	1	influence, influence, influence
12	15	19	33	2	10	7	8	objectivity, objectivity, objective education
0	22	2	3	0	58	2	13	training, training, guidelines and training
12	7	3	7	20	12	7	23	intellectual growth, intellectual growth, intellectual growth
20	20	10	24	1	4	9	0	impartiality, impartiality, impartiality
10	10	3	8	13	17	4	10	academic freedom, academic freedom, academic freedom
0	1	2	2	5	12	7	45	tolerance, political tolerance, tolerance
1	0	4	0	12	13	28	16	Engagement, engagement, engagement
2	1	4	0	13	10	31	7	exposure, exposure, Exposure

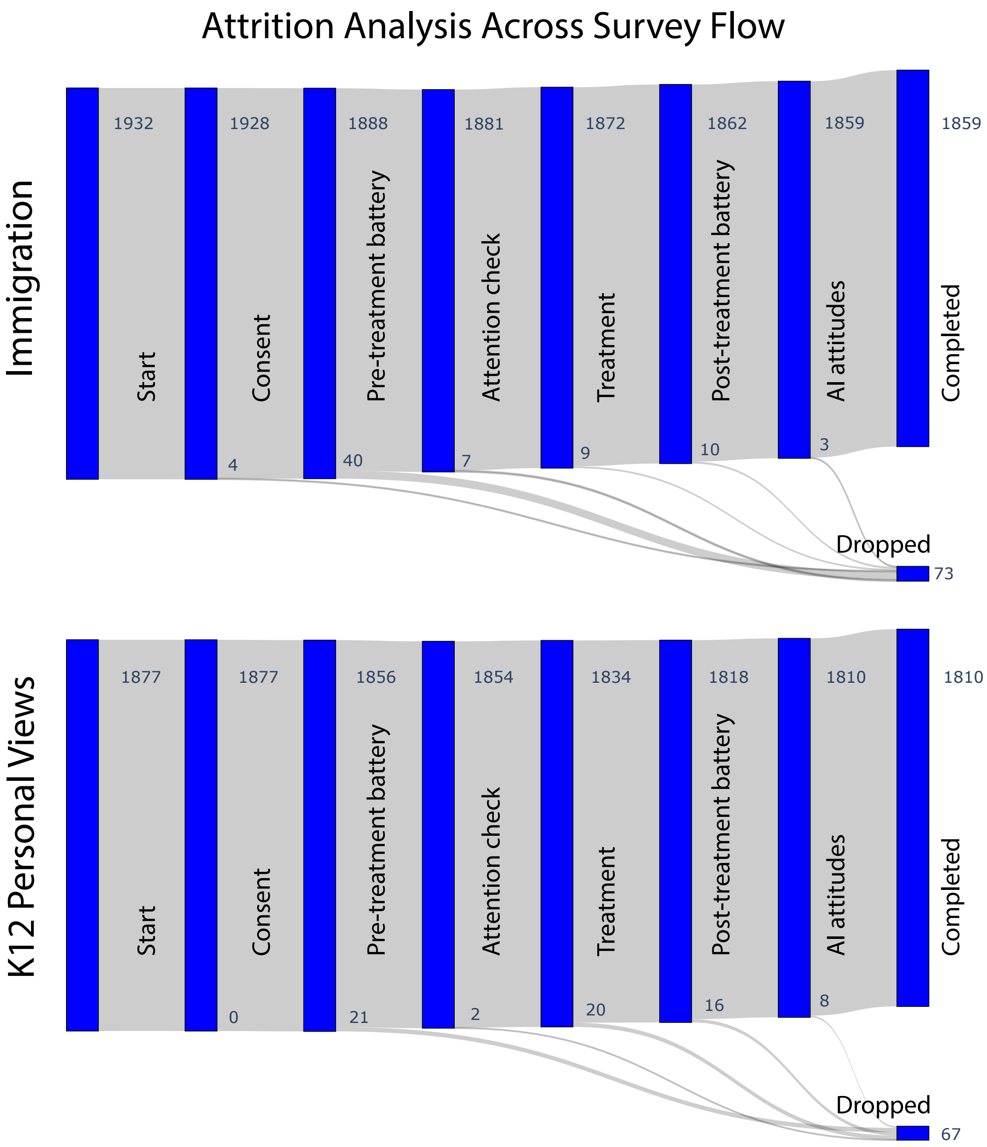

Figure S7: Attrition analysis

9 Attrition analysis across survey flow

Figure S7 shows an attrition analysis of the survey flow. The vast majority of participants completed the surveys: in the Immigration condition, 96.1% of participants completed the survey, and in the K12 condition, 96.3% completed the survey. In both cases, the highest points of exit were the pre- and post- treatment battery of questions, and the AI conversation itself. Only a small number of respondents (9 or 0.5 percent for Study 1 and 20 or 1.1 percent for Study 2) dropped out of the survey at the treatment stage. The number of respondents who dropped out is too low for analysis of attrition; only considering the treatment variables would run tests with 6 different groups on a sample of only 22 for study 1 and 44 for study 2. Given that such tests are likely to be insignificant due to sample size alone, we do not conduct or present them here.

A detailed breakdown of attrition during the treatment is given in Table S10.

Type	Condition	K-12	Immigration
Static	Control	1	0
	Generic	0	1
	Microtargeted	10	1
Dynamic	Dynamic control	2	0
	Motivational interviewing	3	3
	Direct persuasion	2	4

Table S12: Attrition during treatment, broken out by condition.

References

[1] Sorenson, Robinson, Rytting, Shaw, Rogers, Delorey, Khalil, Fulda, and Wingate. An information-theoretic approach to prompt engineering without ground truth labels. *arxiv preprint arXiv:2203.11364*, 2022. URL `https://arxiv.org/abs/2203.11364`.

[2] Xianming Li and Jing Li. Angle-optimized text embeddings. *arXiv preprint arXiv:2309.12871*, 2023. URL `https://arxiv.org/abs/2309.12871`.

[3] Kraskov, Stoegbauer, and Grassberger. Estimating mutual information. 2003. URL `https://arxiv.org/abs/cond-mat/0305641`.

[4] Pei Zhou, Jay Pujara, Xiang Ren, Xinyun Chen, Heng-Tze Cheng, Quoc V. Le, Ed H. Chi, Denny Zhou, Swaroop Mishra, and Huaixiu Steven Zheng. Self-discover: Large language models self-compose reasoning structures. 2024. URL `https://doi.org/10.48550/arXiv.2402.03620`. Cite as: arXiv:2402.03620 [cs.AI] (or arXiv:2402.03620v1 [cs.AI] for this version).

[5] Alexis Palmer and Arthur Spirling. Large language models can argue in convincing ways about politics, but humans dislike ai authors: implications for governance. *Political science*, 75(3):281–291, 2023.

[6] Yidan Yin, Nan Jia, and Cheryl J. Wakslak. Ai can help people feel heard, but an ai label diminishes this impact. *Proceedings of the National Academy of Sciences*, 121(14):e2319112121, 2024. doi: 10.1073/pnas.2319112121.

[7] Yunhao Zhang and Renée Gosline. Human favoritism, not ai aversion: People's perceptions (and bias) toward generative ai, human experts, and human–gai collaboration in persuasive content generation. *Judgment and Decision Making*, 385:e41, 2023.

[8] Thomas H Costello, Gordon Pennycook, and David G Rand. Durably reducing conspiracy beliefs through dialogues with ai. *Science*, 18:e41, 2023.
